# 2D Perovskite Heterojunction‐Based Self‐Powered Polarized Photodetectors with Controllable Polarization Ratio Enabled by Ferro‐Pyro‐Phototronic Effect

**DOI:** 10.1002/advs.202414422

**Published:** 2025-01-22

**Authors:** Xiaoran Yang, Binyi Zhou, Meitong Guo, Yao Liu, Ridong Cong, Leipeng Li, Wenqiang Wu, Shufang Wang, Linjuan Guo, Caofeng Pan, Zheng Yang

**Affiliations:** ^1^ Hebei Key Laboratory of Photo‐Electricity Information and Materials College of Physics Science and Technology Hebei University Baoding 071002 P. R. China; ^2^ Institute of Life Science and Green Development Hebei University Baoding 071002 P. R. China; ^3^ Institute of Atomic Manufacturing Beihang University Beijing 100191 P. R. China

**Keywords:** 2D perovskite film, built‐in field, ferro‐pyro‐phototronic effect, polarization photodetectors, polarization ratio

## Abstract

Metal halide perovskites (MHPs) are commonly used in polarization‐sensitive photodetectors (PDs) for applications such as polarization imaging, remote sensing, and optical communication. Although various methods exist to adjust the polarization‐sensitive photocurrent, a universal and effective approach for continuous control of MHPs’ optoelectronic and polarized properties is lacking. A universal strategy to electrically modulate the polarization ratio (PR) of self‐powered polarized PDs using the ferro‐pyro‐phototronic effect (FPPE) in 2D perovskites is presented. By varying the amplitude and direction of ferroelectric polarization voltage, the built‐in electric field in the heterojunction can be modulated, allowing for controllable PR regulation and adjustable polarization characteristics. Moreover, the polarized pyroelectric photoresponses are realized, significantly enhancing the responsivity, response speed of the polarized PDs. Both the pyroelectric currents and photocurrents exhibit obvious polarization characteristics. This method's versatility is demonstrated by creating three additional quasi‐2D MHP ferroelectric‐based polarized‐sensitive PDs. A proof‐of‐concept for encrypted optical communication is achieved using the UV‐sensitive PDs as light‐sensing units. These findings highlight FPPE's potential to enhance ferroelectric device polarization control, enabling high‐performance and self‐powered polarization photodetection.

## Graphical abstract

A universal strategy to electrically modulate the polarization ratio of self‐powered polarized photodetectors using the ferro‐pyro‐phototronic effect in 2D perovskites is demonstrated. By varying the amplitude and direction of ferroelectric polarization voltage, the built‐in electric field in the heterojunction can be modulated, allowing for controllable polarization ratio regulation and adjustable polarization characteristics, which can be applied for encrypted optical communications.

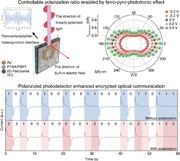

## 1 Introduction

The polarization of reflected or transmitted light helps differentiate materials and structures. Some animals and insects naturally have polarization‐sensitive vision, aiding in prey detection and navigation. This ability has inspired the creation of polarization‐sensitive photodetectors (PDs).^[^ ^1^, ^2^ ^]^ Polarization‐sensitive PDs are gaining attention over conventional ones for applications like polarization imaging, optical radar, remote sensing, object recognition, optical communication, and biomedicine.^[^ ^3^, ^4^, ^5^, ^6^ ^]^ They offer a new degree of freedom by distinguishing and analyzing light's polarization states, enhancing precision and sensitivity in various scenarios.^[^ ^7^, ^8^, ^9^ ^]^ Initial researches on polarization‐sensitive PDs primarily concentrated on macroscopic anisotropy, necessitating intricate patterning and alignment techniques.^[^ ^10^, ^11^ ^]^ On the other hand, anisotropic materials, with low‐symmetry crystal structures like orthorhombic, monoclinic, and triclinic, inherently possess high polarization sensitivity, making them ideal for polarization PDs.^[^ ^12^, ^13^, ^14^, ^15^, ^16^, ^17^ ^]^

Recently, two‐dimensional organic–inorganic hybrid metal halide perovskites (2D MHPs) have shown strong polarization sensitivity due to their parallel layered structures of alternating inorganic slabs and organic spacers,^[^ ^18^, ^19^, ^20^, ^21^ ^]^ highlighting their potential in this area. These atomic‐scale layers along a high‐symmetry axis create a quantum‐confined pattern, naturally imparting anisotropy to the 2D MHPs. Unlike inorganic 2D materials, MHPs can be easily processed into large and low‐cost crystals, enabling the use of their out‐of‐plane anisotropy for polarization‐sensitive photodetection. However, the dynamic adjustment of a polarized perovskite PD with a continuously variable polarization ratio (PR) is seldom reported, despite its potential for multifunctional polarimetric optoelectronics and encrypted optical communication. In the practical application of polarization, PR is basically fixed and cannot be changed. In order to achieve stronger polarization detection capability on the same device, it is necessary to increase PR and improve the device's detection ability for polarized light. In addition, tunable polarized photocurrent could facilitate next‐generation high‐resolution polarimetric imaging.^[^ ^22^, ^23^, ^24^ ^]^ Wang et al. reported a PR‐tunable PD based on 1T′‐MoTe_2_/ambipolar WSe_2_ heterojunction, paving the way for the integration of multiple functional modules into a unified “All‐in‐One” system.^[^ ^23^ ^]^ Various strategies for tunable polarization‐sensitive photocurrent include using metallic nanoantennas on semimetals^[^ ^25^ ^]^ or gate‐tuning band alignment in heterostructures.^[^ ^22^, ^23^ ^]^ However, for perovskites‐based polarized PDs, no researches were found. The aforementioned methods are constrained by strict material design and fabrication complexity, necessitating a simple and effective strategy for achieving continuous control over the optoelectronic and polarized properties in 2D MHPs‐based PDs.

Another strong method for polarized photodetection is to use the built‐in electric field in the heterostructures, in which the photocurrent across the heterojunction interface varies with the angle of polarized light.^[^ ^26^, ^27^ ^]^ Besides, the built‐in electric field at the heterostructure interface can separate photogenerated electron–hole pairs, reducing recombination and enhancing sensitivity in self‐powered polarization‐sensitive PDs. Ferroelectric materials have spontaneous electric polarization that can be altered by an external electric field. Integrating them into heterostructures allows for the electrical tuning of the built‐in electric field by adjusting the ferroelectric polarization voltage and direction.^[^ ^28^, ^29^, ^30^ ^]^ Wu et al. developed a ferroelectric‐doping technique that allows for the detection of linearly polarized light in isotropic 2H‐MoTe_2_.^[^ ^31^ ^]^ The PR can be electrically adjusted by altering the ferroelectric polarization states, transitioning from a positive (unipolar) to a negative (bipolar) regime. Therefore, ferroelectricity enables the creation of electrically switchable anisotropic polarized PDs based on 2D perovskite ferroelectrics. Moreover, ferroelectrics inherently exhibit pyroelectricity. The pyro‐phototronic effect, resulting from the interaction of semiconductor properties, photonic excitations, and the pyroelectric effect, has garnered significant interest for its potential in self‐powered photodetection.^[^ ^32^, ^33^, ^34^, ^35^, ^36^ ^]^ The pyro‐phototronic effect is highly valued in PDs for enhancing responsivity, response speed, and spectral range. Combining it with ferroelectricity‐modulated built‐in electric fields significantly improves polarized photodetection performance with a controllable anisotropic ratio.

This study presents an efficient method for electrically modulating the PR of self‐powered polarized PDs using the ferro‐pyro‐phototronic effect (FPPE) in 2D perovskites. This approach is universally applicable to various 2D perovskite ferroelectrics and different light wavelengths. Cerium ions (Ce^3+^)‐doped 2D perovskite ferroelectric films serve as a key example for UV polarization‐sensitive photodetection. By utilizing a tunable FPPE, we show that the PR in BDA_0.7_(BA_2_)_0.3_EA_2_Pb_3_Br_10_:Ce^3+^ polarization PDs under 320 nm light illumination can be continuously adjusted by varying the ferroelectric polarization voltages, which effectively modulate the built‐in electric field, from 1.9 to 2.8, with a peak responsivity of 2.85 A W^−1^. The mechanisms were identified through electrical measurements and angle‐dependent polarized photodetection. This approach can be applied to various 2D MHPs for continuous electrical modulation of the PR in self‐powered polarization PDs. The tunability of BDAEA_2_Pb_3_Br_10_, BA_2_EA_2_Pb_3_I_10_, and BDA_0.7_(BA_2_)_0.3_MA_4_Pb_5_I_16_‐based polarized PDs working under 360, 532, and 785 nm was assessed. These findings offer a strategy for creating high‐performance, self‐powered polarization PDs using MHPs, serving as a valuable resource for other ferroelectric material‐based polarized devices.

## 2 Results and Discussions

### 2.1 Electrically Tunable Polarization Photodetection Characteristics Induced by the Ferroelectric Effect

**Figure 1:**
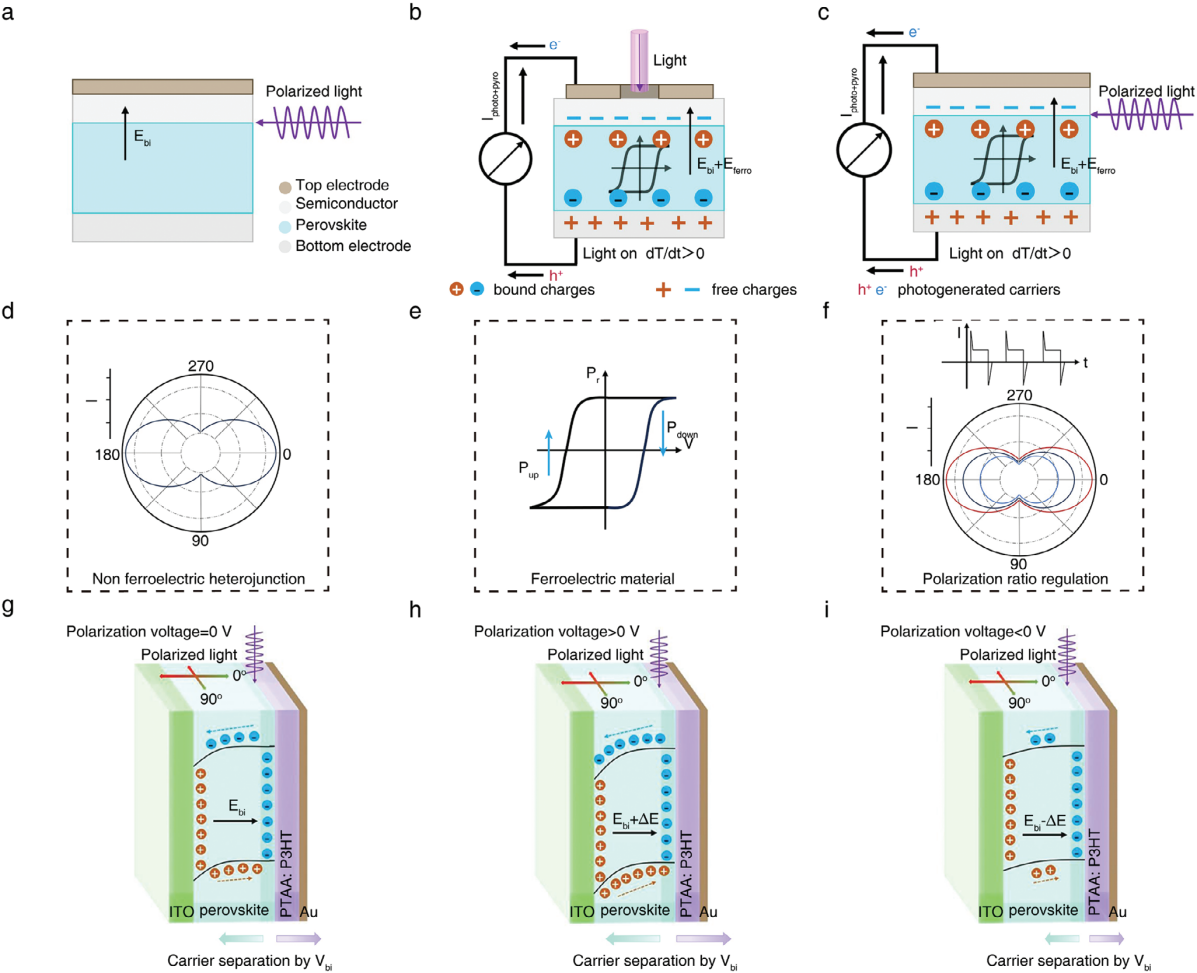
Schematic diagram of the working mechanisms of perovskite‐based polarization sensitive PDs. Schematic diagram of a) non‐ferroelectric perovskite‐based heterojunction, ferroelectric perovskite‐based heterojunction b) when light is incident perpendicular to the interface, and c) when light is parallel to the interface. Schematic diagram of d) polarization characteristics of non‐ferroelectric perovskite‐based heterojunction, e) ferroelectric hysteresis loop of the ferroelectric perovskite, and the FPPE induced‐polarization characteristics with controllable PR. The energy band diagrams of our PDs when g) without applying ferroelectric polarization voltage, h) applying a positive ferroelectric polarization voltage, and i) applying a negative ferroelectric polarization voltage.

The heterojunction's built‐in electric field is a promising tool for polarization detection due to its efficient carrier transport and separation, enhancing polarization sensitivity.^[^ ^26^ ^]^ However, built‐in electric fields are typically fixed, and the polarization photodetection mechanism remains unclear. Ferroelectric materials, a type of dielectric, have switchable, nonvolatile polarization that can easily adjust various physical properties of low‐dimensional materials, like band structure, carriers, and optical conductivity. 2D MHP ferroelectrics are ideal candidate to create heterojunctions for polarized devices. **Figure** **1** compares the working mechanisms of the different polarization PDs based on perovskites. In Figure 1a, heterojunctions with non‐ferroelectric perovskites show a built‐in electric field (*E*_bi_) in the depletion region. This occurs due to energy level realignment to match the Fermi levels, causing band bending and an electric field directed from the perovskite to the other semiconductor. When polarization direction of the incident light is aligned with or against the built‐in electric field, photogenerated carriers are produced and separated, resulting in anisotropic photoresponse (Figure 1d). Traditional heterojunctions lack dynamic regulation of the *E*_bi_, thus with fixed PR value. 2D MHP ferroelectrics, with adjustable spontaneous polarizations and inherent pyroelectric properties, are ideal alternatives. To obtain devices with controllable PR, it is necessary to dynamically control the value and direction of the *E*_bi_ in the device, altering the photoresponses of the device under different angles of polarized light irradiation. Our group has demonstrated a series of 2D MHPs‐based self‐powered PDs boosted by FPPE.^[^ ^37^, ^38^, ^39^, ^40^, ^41^ ^]^ Figure 1b illustrates the FPPE mechanism in 2D MHP ferroelectric‐based PDs. Light exposure generates hot carriers in the MHP, raising lattice temperature and reducing spontaneous polarization. This causes compensating charges and photogenerated carriers to flow, producing positive pyroelectric and photocurrents (*I*_photo+pyro_). When the light is off, lattice cooling generates negative pyroelectric currents. Figure 1d illustrates that the ferroelectric domain provides a residual field, influencing charge distribution at the interface and altering the electric field strength. This enables dynamically adjustable pyro‐phototronic photoresponses. When polarized light hits parallel to the heterojunction interface (Figure 1c), the anisotropy between the light and electric field allows for polarization photodetection. When in a polarized up (*P*_up_) or down (*P*_down_) state, 2D MHPs can create a negative or positive ferroelectric field to adjust the overall electric field. Consequently, the ferroelectric‐induced built‐in electric field (*E*_ferro_) and the heterojunction's intrinsic built‐in electric field (*E*_bi_) collaborate to produce polarization PDs with adjustable anisotropic ratios based on FPPE (Figure 1f). Combining ferroelectricity and pyroelectricity significantly enhances detectivity (D^*^), responsivity (*R*), and response speed, greatly boosting the performance of polarization‐sensitive devices. Energy band diagrams in Figure 1g–i clarify how our PR‐tunable PDs operate. We used a polarizer and half‐wave plate to control the incident laser's polarization from 0° to 360°, with 0° defined as light polarized parallel to the heterojunction interface. The ferroelectric effect in the out‐of‐plane direction is relevant due to the device's sandwich structure and out‐of‐plane photoresponse measurements. Under unpoled conditions (Figure 1g), only *E*_bi_ is produced due to Fermi level alignment and band bending. The built‐in electric field drives electrons to the perovskite and holes to the transport layer. When polarized light aligns with *E*_bi_, photogenerated hot carriers achieve maximum momentum, enhancing separation and minimizing recombination, resulting in peak *I*_pyro_ and *I*_photo_. When polarized light is vertical to *E*_bi_, the exciton separation/transport becomes weak, resulting in minimum *I*_pyro_ and *I*_photo_. Applying a positive ferroelectric polarization voltage aligns the electric dipoles in the perovskite ferroelectric film with the external field, strengthening the device's built‐in electric field (*E*_bi_ + Δ*E*) and causing an upward bend in the energy band (Figure 1h). This enhanced electric field facilitates the separation and transport of photogenerated carriers, reduces recombination, and boosts the photodetector's performance. As shown in Figure 1i, applying a reverse ferroelectric polarization voltage decreases the PD's electric field (*E*_bi_ − Δ*E*) due to residual polarization. This causes the energy band to bend downward, hindering photogenerated carrier separation and transport, thus reducing the PD's PR. The ferroelectrics' polarization intensity and direction can be easily adjusted with an external voltage, allowing continuous and controllable modulation of the heterojunction's *V*_bi_ and the PR of polarization‐sensitive PDs.

### 2.2 Characterization of the 2D Perovskite and the Resultant PDs

The detailed analysis of 2D perovskite ferroelectrics and their PDs is thoroughly discussed. Ce^3+^‐doped 2D wide bandgap ferroelectric perovskite films are highlighted as a key example for polarization‐sensitive UV photodetection, which is used in fields like communication, imaging, remote sensing, and military surveillance. Figure S1 (Supporting Information) illustrates the fabrication of 2D MHP films using a one‐step heating spin‐coating method, conducted at a stable temperature of 70 °C under ambient conditions (24 °C, 35 ± 10 RH%), thereby obviating the necessity for the glove box. In order to enhance the absorbance of the film in the ultraviolet region,^[^ ^42^, ^43^ ^]^ Ce^3+^ were introduced into our early reported 2D perovskite BDA_0.7_(BA_2_)_0.3_EA_2_Pb_3_Br_10_^[^ ^38^ ^]^ at varying concentrations. Based on the top‐view scanning electron microscopy (SEM) images presented in Figure S2 (Supporting Information), the film morphologies exhibit favorable characteristics following the doping process. Figure S3a (Supporting Information) shows X‐ray diffraction (XRD) patterns for six films with varying doping levels, highlighting sharp peaks at 15° and 30.3°, linked to the (111) and (202) crystal planes. Figure S3b–d (Supporting Information) presents the UV–visible absorption and photoluminescence (PL) spectra, revealing that Ce^3+^ doping boosts light absorption below 350 nm, peaking at 5% Ce^3+^. In addition, Ce^3+^ doping causes a blueshift in the PL peak, without impairing the phase purity. To explore fluorescence dynamics in perovskite films, time‐resolved photoluminescence (TRPL) measurements were performed (see Figure S3e, Supporting Information). The TRPL curves were fitted with a double exponential decay model to determine photoluminescence lifetimes, with shorter lifetimes indicating surface recombination and longer ones indicating bulk recombination. As shown in Table S1 (Supporting Information), the film with 5% Ce^3+^ doping has a longer lifetime, implying fewer defects and better film quality. Subsequently, the trap densities in the six 2D perovskite films were assessed using the space charge‐limited current (SCLC) method. The dark current–voltage (*I–V*) characteristics of the electron‐only and hole‐only devices were measured (Figure S4, Supporting Information). Figure S3f (Supporting Information) shows the calculated trap densities for holes and electrons for the six films based on the capacitance–voltage (*C–V*) measurements plotted in Figure S5 (Supporting Information). Perovskite film with 5% Ce^3+^ shows a much lower trap density (3.2 × 10^14^ cm^−3^ for electrons and 5.8 × 10^14^ cm^−3^ for holes) than other films (Table S2, Supporting Information), meaning fewer nonradiative recombination rates. The 5% Ce^3+^doped perovskite film showed improved crystallinity, lower trap density, optimal orientation, and high phase purity. Grazing‐incidence wide‐angle X‐ray scattering (GIWAXS) measurements, shown in Figure S6 (Supporting Information), confirmed sharp elliptical signals, indicating enhanced crystallinity and vertical alignment, which supports efficient carrier transport between electrodes. An in‐depth elemental analysis was performed using energy‐dispersive spectroscopy (EDS), showing uniform distribution of Ce, Br, and Pb in the film (Figure S7, Supporting Information). X‐ray photoelectron spectroscopy (XPS) confirmed Ce incorporation into the perovskite film by substituting larger Pb^2+^ with smaller Ce^3+^, as shown in the XPS spectra for Pb^2+^, Br^−^, and Ce^3+^ (Figure S8, Supporting Information). Piezoresponse force microscopy (PFM) was also used to evaluate ferroelectric properties. Figure S9d (Supporting Information) shows ferroelectric domain polarization switching, demonstrated by a hysteresis and butterfly loop via out‐of‐plane PFM. The topography of the 5% Ce^3+^ doped perovskite film in Figure S9a (Supporting Information) shows a smooth, pinhole‐free surface. Figures S9b and S8c (Supporting Information) display amplitude and phase images from vertical PFM mode, with color contrast and 180° phase differences indicating different polarization orientations in the out‐of‐plane domains. Atomic force microscopy (AFM) images in Figure S10 (Supporting Information) reveal that Ce^3+^ doping smoothens the film's surface.

**Figure 2:**
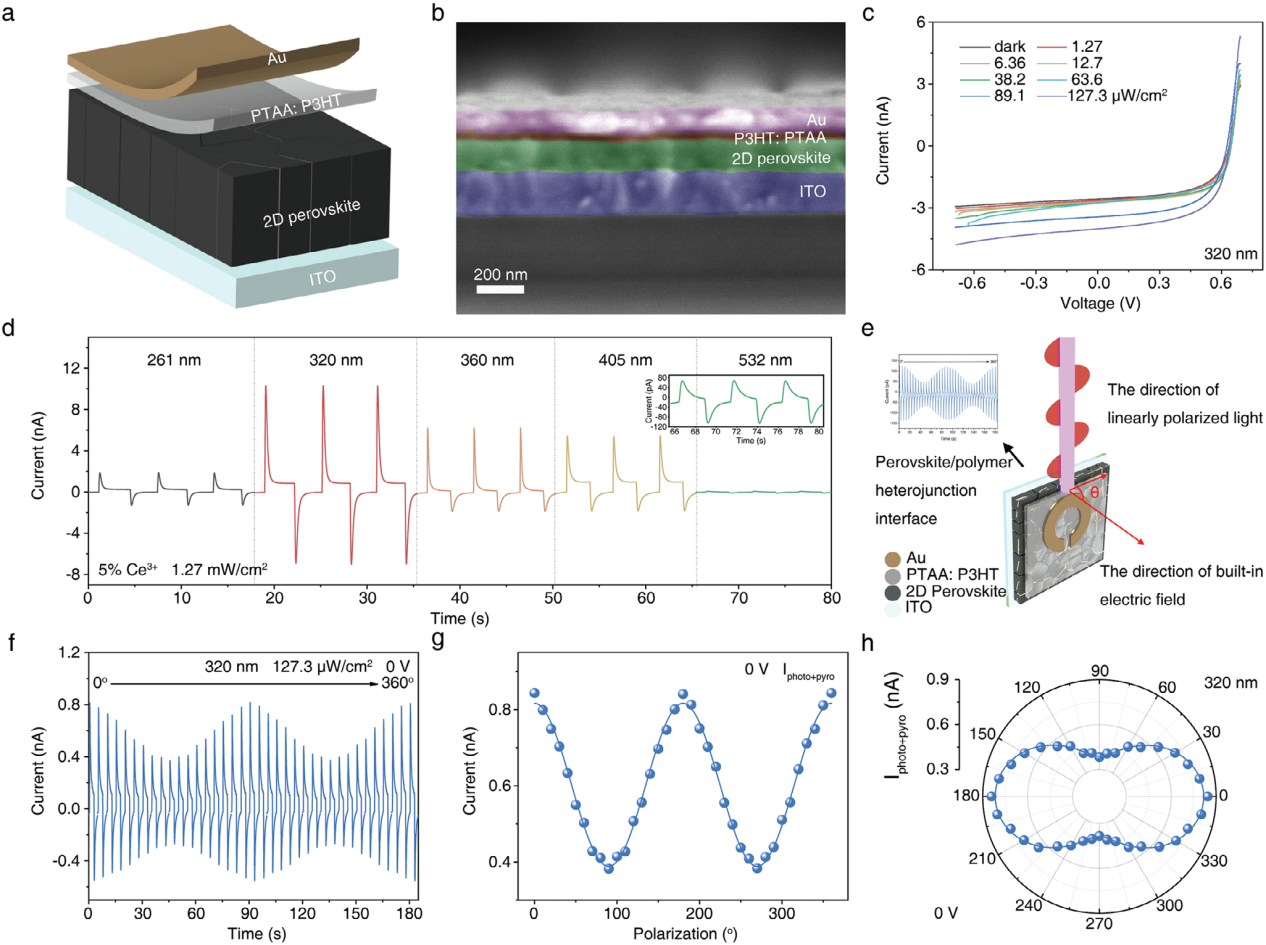
The UV polarized photodetection performances of the 5% Ce^3+^ doped perovskite film‐based PD. a) The structural diagram and b) cross‐sectional SEM image of 5% Ce^3+^ doped perovskite film‐based PD. c) *I–V* characteristics of the PD when illuminated with a 320 nm laser at different power densities ranging from 0 to 127.3 µW cm^−2^. d) The self‐powered spectral photoresponse of 5% Ce^3+^ doped perovskite film‐based PD when illuminated with 261, 320, 360, 405, and 532 nm laser irradiations. e) Schematic diagram of polarized sensitive PDs’ operating mode. f) Photoresponses of the PDs under 320 nm laser with different polarization angles under zero bias voltage. g,h) Polarization dependence of the angle‐resolved *I*_photo+pyro_ under 320 nm polarized laser.

A self‐powered PD was then fabricated using an ITO/2D perovskite/PTAA: P3HT/Au structure. **Figure** **2**a,b illustrates the PD's schematic and cross‐sectional SEM image with a 5% Ce^3+^ doped film. PTAA: P3HT was chosen as the hole transport layer for its high UV transmittance (over 80% between 230 and 350 nm, Figure S11, Supporting Information). Consequently, 261 and 320 nm lasers were directed onto the PDs’ top side. The optical image of the device is shown in Figure S12 (Supporting Information), with an active area of 0.00785 cm^2^. The spectral responses of the PDs in the ultraviolet range showed significantly improved UV responses between 260 and 320 nm after Ce^3+^ doping (Figure S13, Supporting Information). Since the PD had the strongest response at 320 nm (Figure 2d), this wavelength was chosen as the main light source. In addition, we evaluated the light induced temperature changes of the 5% Ce^3+^ film using a thermal imaging camera. Figure S14 (Supporting Information) shows the thermal images of the 5% Ce^3+^ film illuminated with 261, 320, 360, 405, and 532 nm lasers, respectively. All images show the temperature increase with increasing laser irradiation power. As shown in Figure S15 (Supporting Information), under the same power (10 mW) laser irradiation, the temperature change at 320 nm is 0.56 °C, which is much higher than other wavelengths. Besides, the trend of temperature variation versus laser wavelength is consistent with the spectral photoresponses in Figure 2d, proving that the pyro‐phototronic effect causes the current spikes of the PDs. Figure 2c displays the *I–V* characteristics of the 5% Ce^3+^ doped perovskite film‐based PD, tested in dark and under 320 nm laser at power densities from 0 to 127.3 µW cm^−2^, showing clear rectifying behavior and high open‐circuit voltage (*V*_oc_). Figure S16a (Supporting Information) shows a typical four‐stage pyroelectric photoresponse explained by the FPPE (Figure S16b, Supporting Information). Without light, only dark current was present in the external circuit. When the laser was activated, electron–hole pairs were generated by photoexcitation and directed by the built‐in electric field, creating photocurrent (*I*_photo_). Light absorption also caused a photothermal effect, raising the temperature of the PDs and reducing the ferroelectric polarization intensity in the 2D ferroelectric perovskite film, leading to charge redistribution and generating a positive pyroelectric current (*I*_pyro_). The total output current is *I*_photo+pyro_. At a constant temperature, ferroelectric polarization is stable, producing only a photocurrent. When the laser is turned off, PDs cool, increasing ferroelectric polarization and generating a reverse pyroelectric current (*I*_pyro‘_), while the photocurrent ceases, leaving only dark current. The performance metrics of two PDs with 0% and 5% Ce^3+^ doped perovskite films were analyzed based on the FPPE. Figure S17 (Supporting Information) displays the *I–V* curves of both PDs under dark and light conditions, showing that the 5% Ce^3+^ doped perovskite film‐based PD has higher *V*_oc_ and photocurrents. Figure S18 (Supporting Information) shows that the 5% Ce^3+^ doped perovskite film‐based PD has the fastest response time at 50 Hz under a 320 nm laser with a power density of 1.27 mW cm^−12^, with rise and fall times increasing by 83.2 µs and decreasing by 617.7 µs, respectively, compared to the 0% Ce^3+^ doped perovskite film‐based PD (Figure S19, Supporting Information). Overall performance data for the 0% and 5% Ce^3+^ doped perovskite film‐based PDs under dark conditions and the laser illuminations are presented in Figures S20–S22 (Supporting Information). Figure S22a (Supporting Information) illustrates that the 5% Ce^3+^ PD's on‐off photoresponses at 0 bias under a 320 nm laser, with power densities from 0.12 to 127.3 µW cm^−2^, show transient pyroelectric currents. Figure S22b (Supporting Information) plots *I*_photo+pyro_‐*I*_pyro″_, *I*_photo+pyro_, and *I*_photo_ from Figure S22a (Supporting Information) against power density, revealing that all output currents increase monotonously with power density. Photocurrents exhibit a nearly linear response over the range, contrasting with the power density‐dependent increase in pyroelectric currents. The *R* and D* values were calculated and plotted in Figure S22c,e (Supporting Information) as a function of power density, with equations provided in the supporting information. Both *R* and D* decrease rapidly with increasing power density due to higher charge recombination rates at greater illumination intensity. The maximum values are 2.85 A W^−1^ for *R* and 4.38 × 10^20^ Jones for D*, achieved at the lowest intensity of 0.12 µW cm^−2^. Also, the overall performances of the 0% Ce^3+^ doped perovskite film‐based PD toward 320 nm laser were measured and plotted in Figure S23 (Supporting Information). In comparison to the 0% Ce^3+^, the 5% Ce^3+^ doped perovskite film‐based PD exhibited significant enhancement for 320 nm laser. Also, we evaluated the impact of several factors like operating temperature, bias voltage, and light switching frequency on the performances of the PDs, since they can influence the ferro‐pyro‐phototronic effect. The corresponding results are shown in Figures S24–S26 (Supporting Information), respectively. Figure S24 (Supporting Information) shows the temperature dependence of ferroelectric pyroelectric photoelectronic effects in PD based on 5% Ce^3+^ doped thin films. As the operating temperature increases, the output photocurrent and pyroelectric current continue to decrease. Figure S25a (Supporting Information) shows that when the positive bias voltage exceeds 1.2 V, the negative pyroelectric current will disappear. As shown in Figure S26 (Supporting Information), when the light switching frequency is changed, the photocurrent plateau remains basically unchanged, and the pyroelectric current does not disappear. The polarization characteristics of the PDs without applying a ferroelectric polarization voltage were analyzed. Figure 2e and Figure S27 (Supporting Information) illustrate the operating mode and testing setup for polarized sensitive PDs. The self‐powered polarization characteristics were tested using a 320 nm laser (Figure 2f–h). A half‐wave plate was used to adjust the incident light angle from 0° to 360°, with 0° parallel to the PD's built‐in electric field. The *I*_photo+pyro_ showed a PR of 2.13. For *I*_photo_ and *I*_pyro_, the PR are 1.65 and 2.28, respectively. Polarization testing of the PD with a 261 nm laser revealed significant polarization properties in the solar‐blind UV region, showing PR of 1.98, 1.62, and 2.16 for *I*_photo+pyro_, *I*_photo_, and *I*_pyro_, respectively. These results indicate the PD's capability to detect polarized light in the UV band, including the solar‐blind range.

### 2.3 Polarized‐Photodetection Behaviors with Controllable PR

**Figure 3:**
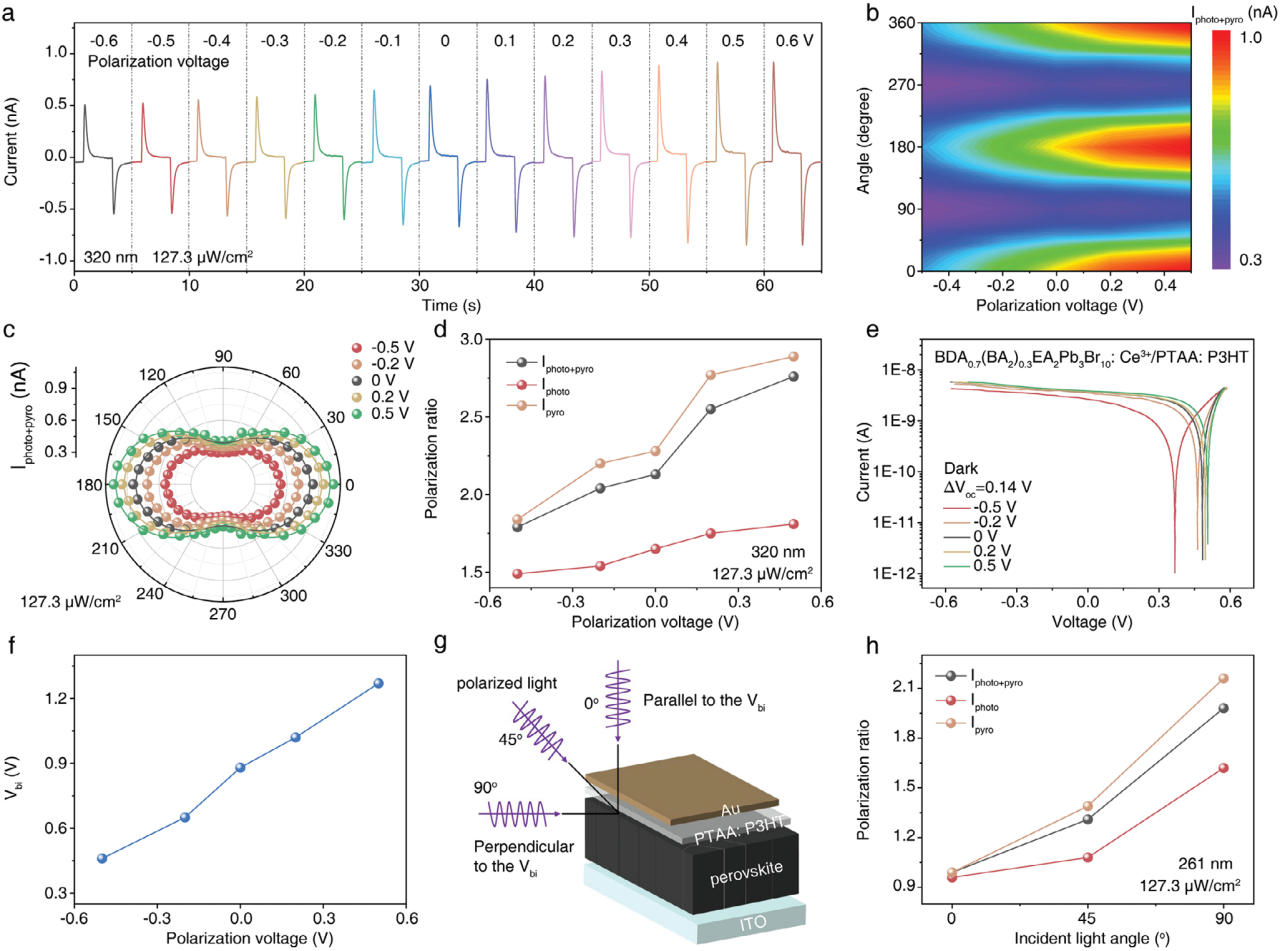
The regulation of 5% Ce^3+^ doped perovskite film‐based PDs’ polarization ratios by regulating residual polarizations. a) The zero bias photoresponses of the PD towards 320 nm laser after been applying with different ferroelectric polarization voltages. b) Polarization‐dependent current (*I*_photo+pyro_) mapping at different ferroelectric polarization voltages under illumination at 320 nm (127.3 µW cm^−2^). c) Polar diagram of the polarization‐sensitive *I*_photo+pyro_ under five ferroelectric polarization voltages at light wavelength of 320 nm, 127.3 µW cm^−2^. d) Polarization voltage‐dependent PR of *I*_photo+pyro_, *I*_photo_, and *I*_pyro_ at light wavelength of 320 nm with a power density of 127.3 µW cm^−2^. e) Dark *I–V*, showing the change of *V*_oc_, f) *V*_bi_ of the PDs. g) Schematic diagram of heterojunction polarized PDs illumination with different incidence angles. h) The incident angle dependent PR of *I*_photo+pyro_, *I*_photo_, and *I*_pyro_.

To study how ferroelectric polarization voltages affect photoresponse, we applied various ferroelectric polarization voltages to our PDs before the photoresponses measurements and used a 320 nm laser with a power density of 127.3 µW cm^−2^. The device's response increased with ferroelectric polarization voltage from −0.5 to 0.5 V (**Figure** **3**a), due to the enhanced built‐in electric field. Figure 3b illustrates how the polarization‐dependent current mapping of our PDs varies with different polarization bias voltages under 320 nm linearly polarized laser illumination, showing that *I*_pyro+photo_ is significantly influenced by the polarization angle and ferroelectric polarization voltage. To effectively demonstrate the impact of various ferroelectric polarization voltages on PD polarization performance, polar diagrams of the polarization‐sensitive output currents under five ferroelectric polarization voltages were created (Figure 3c,d, and Figure S28, Supporting Information). The intensities of *I*_photo+pyro_, *I*_photo_, and *I*_pyro_ change periodically with polarization angle. As ferroelectric polarization voltage rises, the momentum of hot carriers excited by linearly polarized light shifts directionally, peaking at 0° and 360° and dipping at 90° and 270°. Consequently, the PRs of *I*_photo+pyro_, *I*_photo_, and *I*_pyro_ increase with higher ferroelectric polarization voltage. For example, the PR of *I*_pyro_ can be tuned from 1.9 to 2.8. In addition, the impact of laser power on the PR of PDs is examined. As shown in Figures S29 and S30 (Supporting Information), the modulation method of PR by applying different ferroelectric polarization voltages is applicable under different power densities. Also, our PDs have the ability to regulate the PRs in the solar‐blind ultraviolet band. As shown in Figures S31–S35 (Supporting Information), a positive correlation was observed between the ferroelectric polarization voltage and the PR of the three currents (*I*_photo+pyro_, *I*_photo_, and *I*_pyro_).

To investigate if changes in PR relate to variations in *V*_bi_, we firstly measured the dark *I*–*V* curves of the PD at different ferroelectric polarization voltages (Figure 3e) since the *V*_oc_ is highly dependent on the *E*_bi_. The *V*_oc_ increase with the increase of the ferroelectric polarization voltages in the dark. The Δ*V*_oc_ is 0.14 V when the ferroelectric polarization voltage changes from −0.5 to 0.5 V. We further examined the effect of ferroelectric polarization voltage on *V*_bi_ through *C–V* tests (Figure S36, Supporting Information). By performing linear fitting of *C*(*V*)^−2^ versus the external applied bias (V), the *V*_bi_ can be obtained. As shown in Figure 3f, the *V*_bi_ of the PDs rises from 0.88 V (unpolarized condition) to 1.02 V with a ferroelectric polarization voltage of 0.2 V, and further to 1.27 V with a ferroelectric polarization voltage of 0.5 V. For reverse polarization conditions, the *V*_bi_ decreased to 0.65 and 0.46 V, with ferroelectric polarization voltages of −0.2 and −0.5 V, respectively. The above results suggest that by changing the ferroelectric polarization voltage and direction, the *V*_bi_ of the devices can be effectively modulated, thus regulating the PR of the polarized sensitive PDs. To further prove that the interaction between built‐in electric field and polarized light induce the polarized photoresponses, the incidence angle‐dependent polarization performances of the PDs were evaluated, using 261 nm polarized light. Here, the incidence angle means the angle between the propagation direction of light and the direction of *V*_bi_. As shown in Figure 3g, 0° indicates that the propagation direction of light is parallel to *V*_bi_, while 90° denotes the vertical relationship. As shown in Figure S37a,b (Supporting Information), no polarization effect were observed with light incidence angle of 0°. For 45° condition, the output current of PDs slightly varies with polarization angles (Figure S37c,d), indicating weaker polarization characteristics than the 90° condition. Figure 3h illustrates the variations in the PR of *I*_photo+pyro_, *I*_photo_, and *I*_pyro_ as a function of the incidence angle. As the incident angle increases, the PR of all three currents significantly rises, indicating that the polarization characteristics of heterojunction devices are linked to the incident angle of polarized light. When the polarized light travels parallel to *V*_bi_, the photogenerated hot carriers have uniform momentum across all polarization directions, showing no polarization characteristics (Figure S38a, Supporting Information). However, as the angle between the light's propagation direction and *V*_bi_ increases, the device's PR also increases, reaching a maximum value when the polarized light is perpendicular to *V*_bi_. For 45° condition (Figure S38b, Supporting Information), the polarized light possesses a polarized component oriented in the 90° direction, resulting in a moderate polarized PR due to the limited interaction between the polarized light and built‐in electric field. For 90° condition, *V*_bi_ maximizes the photogenerated carriers’ momentum along the electric field, enhancing electron–hole separation, reducing recombination, and leading to maximum currents and highest PR. This indicates that the vertical strength of the *V*_bi_ component relative to the polarized light's propagation direction is key to the PR in our 2D MHP ferroelectric‐based heterojunctions.

### 2.4 The Universality of the PR Regulation Strategy

**Figure 4:**
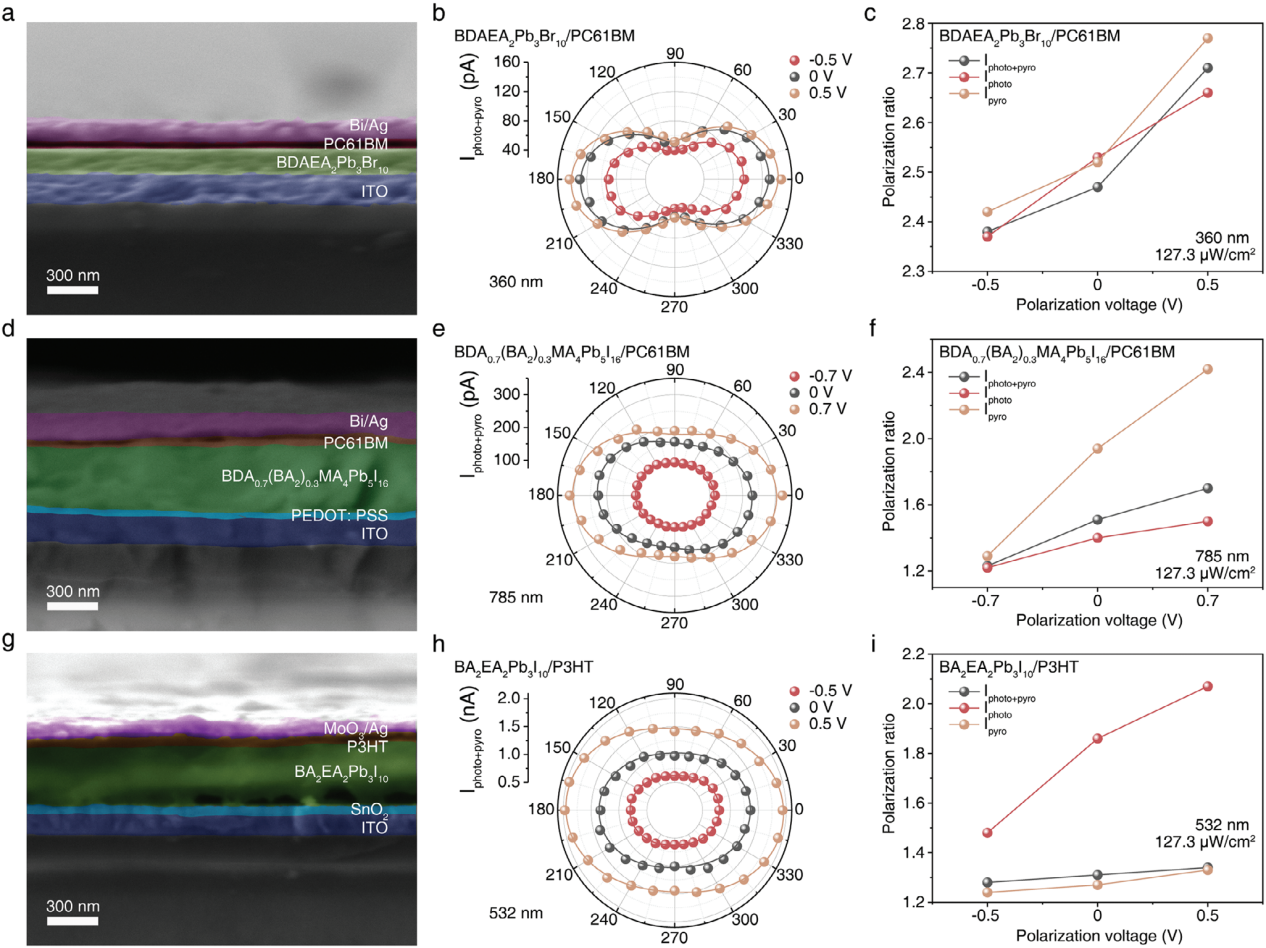
Universality of the PR regulation strategy using another three PDs. Cross‐sectional SEM images of the PD based on a) BDAEA_2_Pb_3_Br_10_ film, d) BDA_0.7_(BA_2_)_0.3_MA_4_Pb_5_I_16_ film, and g) BA_2_EA_2_Pb_3_I_10_ film. Polar diagrams of the polarization‐sensitive *I*_photo+pyro_ at zero bias for b) BDAEA_2_Pb_3_Br_10_ film, e) BDA_0.7_(BA_2_)_0.3_MA_4_Pb_5_I_16_ film, and h) BA_2_EA_2_Pb_3_I_10_ film based PDs. Polarization voltage dependent PR of *I*_photo+pyro_, *I*_photo_, and *I*_pyro_ for PD based on c) BDAEA_2_Pb_3_Br_10_ film, f) BDA_0.7_(BA_2_)_0.3_MA_4_Pb_5_I_16_ film, and i) BA_2_EA_2_Pb_3_I_10_ film.

To validate the universality of our PR regulation strategy via applying ferroelectric polarization voltage with different amplitude and directions, another three quasi‐2D MHP ferroelectrics we previously reported were prepared and fabricated into heterojunctions, namely BDAEA_2_Pb_3_Br_10_,^[^ ^33^ ^]^ BDA_0.7_(BA_2_)_0.3_MA_4_Pb_5_I_16_ coupled with (TMIM)PbI_3_,^[^ ^31^ ^]^ and BA_2_EA_2_Pb_3_I_10_.^[^ ^36^ ^]^ Figure S39a–f (Supporting Information) illustrates the characterizations of above three MHP films, all of which exhibit excellent morphology, crystallinity, and high phase purity. Besides, the absorption cut‐off edges of the above three films locate at 450, 780, and 600 nm, beneficial for achieving absorption of light in different wavelength bands. Based on this, three kinds of polarized‐sensitive PDs with following structures were fabricated: ITO/BDAEA_2_Pb_3_Br_10_/PC61BM/Bi/Ag, ITO/PEDOT: PSS (4083)/BDA_0.7_(BA_2_)_0.3_MA_4_Pb_5_I_16_/PC61BM/Bi/Ag, and ITO/SnO_2_/BA_2_EA_2_Pb_3_I_10_/P3HT/MoO_3_/Ag. **Figure** **4**a,d,g presents SEM cross‐sectional images of the three types of PDs, all showing multilayer sandwich structures with distinct interfaces. First, we assessed the photoresponses of three PDs under different ferroelectric polarization voltages (Figures S40, S42, and S44, Supporting Information), from which the optimal ferroelectric polarization voltages can be obtained. Then, we tested their polarization characteristics using polarized light at wavelength of 360, 785, and 532 nm (Figures S41, S43, and S45, Supporting Information), with laser directions perpendicular to the heterojunctions' built‐in electric fields. Figure 4b,e,h shows the *I*_photo+pyro_ polar coordinates of the PDs under these conditions. All PDs behave obvious polarization adjustable characteristics. As shown in Figure 4c,f,i, it can be seen that as the ferroelectric polarization voltage increases, the PR of the three currents of three PDs also increases, illustrating the effective and dynamic modulation of the polarization characteristics. Then, we examined the dark *I–V* curves of three PDs in the dark after applying with different ferroelectric polarization voltages (Figure S46, Supporting Information). As shown in the figure, the Δ*V*_oc_ values of BDAEA_2_Pb_3_Br_10_, BA_2_EA_2_Pb_3_I_10_, and BDA_0.7_(BA_2_)_0.3_MA_4_Pb_5_I_16_ are 0.11, 0.15, and 0.17 V, respectively, indicating that the regulation in PR is due to the modulation of built‐in electric field by ferroelectric polarization. The above results demonstrated that the strategy of dynamically regulating PR through applying ferroelectric polarization voltage is applicable to heterojunction PDs with different structures and response bands. Although the PR values for our devices are low, the devices can be prepared without complex processes and can be switched between non polarized or polarized ones. In the future, to improve the RP value of the perovskite ferroelectrics‐based devices, methods such as growing single crystals and constructing single crystal heterojunctions with enhanced crystallinity and built‐in field may be employed.

### 2.5 Optical Communication Applications of the Polarized‐Sensitive PDs

**Figure 5:**
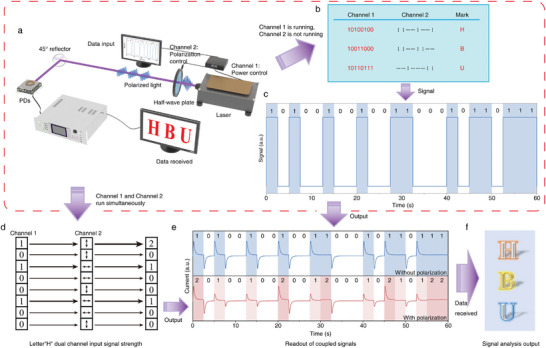
Optical communication applications of the polarized‐sensitive PDs. a) Diagram showing how optical communications use polarization‐dependent PDs by utilizing both light intensity and polarization state as separate information carriers. b) Diagram of the optical signal (HBU, 10100100101100110110111) and polarization signal from the transmitter, with horizontal arrows for 90° polarized light and vertical arrows for 0° polarized light. c) Corresponding photocurrents of the encrypted commands when only Channel 1 is working. d) Schematic illustration of the emitted light signals from the laser and the modulation of the polarization states when both Channel 1 and Channel work. e) Output current of the PD when signal “101001001010011011111” is transported without (top) and with polarization state (bottom).

Unlike traditional polarization‐insensitive PDs that only detect light intensity, polarization PDs can also identify polarization direction. This allows new optical communication systems to potentially transmit data more efficiently by using polarization as a carrier. This advancement has led to the development of encrypted optical communication using polarization‐sensitive ultraviolet PDs. **Figure** **5**a illustrates the operational mechanism, where optical switch and polarization information are transmitted via Channel 1 and Channel 2, respectively. “1” is indicated by turning on linearly polarized light, and “0” by turning off the signal sent to it. For 2D perovskite ferroelectric thin film heterojunction polarized PDs, a vertical arrow denotes 0° polarized light. At the receiving end, a polarization‐sensitive heterojunction PD detects linearly polarized light carrying binary signals. To simulate wireless data transmission (Figure 5b), each letter of “HBU” is converted into ASCII code and polarized light signals. The signal is sent to the PD without using Channel 2, resulting in on/off light signals for “HBU” (Figure 5e, without polarization). Activating Channel 2 allows both channels to transmit signals simultaneously. Figure 5d illustrates the combined modulation output of Channels 1 and 2 for the letter “H”. Three sets of ASCII codes and polarization signals are processed together, resulting in the output shown in Figure 5e (with polarization). The figure illustrates dual‐channel modulation output (201001002001200102122) and explains encoding encryption. When the transmission signal is “1” and the polarization state is 0°, the dual channel signal is “11” with a maximum optical output of “2”. If the polarization state is 90°, then the signal is “10” with a reduced optical output of “1”. When the transmission signal is “0”, the output signal is also “0”, regardless of the polarization state. Thus, for dual‐channel signals “01” or “00”, the output remains “0”. The output signal can be decoded at the receiving end using a key based on the polarization angle. If the output signal is “2”, then it indicates that the dual‐channel transmission data is “11”, with channel 1 carrying the transmitted signal. Therefore, the first digit “1” is taken as the transmission signal. Similarly, other output signals can be analyzed. When the output signal is “1”, the transmission signal is “1”, and when the output signal is “0”, the transmission signal is “0”. Analyzing this output reveals the letter “HBU” (Figure 5f). To investigate the use of dynamically adjusting PR values in optical communication, we encoded the ASCII for “HBU” using polarization angles of 0°, 30°, 60°, and 90° as keys (0° = 4, 30° = 3, 60° = 2, 90° = 1). We tested polarized light transmission at voltages of 0.5 and −0.5 V. As shown in Figure S47 (Supporting Information), the signal has five states, and data transmission is impossible without the key, indicating enhanced encryption compared to Figure 5e. Figure S47b (Supporting Information) shows that the output signal with a polarization voltage of 0.5 V differs more significantly from that at −0.5 V, enhancing signal disparity, resolution, and transmission accuracy. Currently, we use binary encoding, which poses a decryption risk; thus, we are changing to ASCII encoding for transmission, as indicated in Figure 5b. Currently, we use binary encoding for transmission, which poses a risk of decryption. To enhance security, we are switching to ternary encoding (Figure S48a, Supporting Information), resulting in a transmission signal with four states (Figure S48b, Supporting Information). This approach significantly reduces the risk of decryption compared to the polarized binary signal in Figure 5e. Thus, using higher base encoding improves the security of signal transmission. The data highlight that polarization‐sensitive PDs allow for encrypted, multichannel transmission, maintaining readout speed, and improving data resolution.

### 2.6 Stability and Repeatability of the PDs

The stability of perovskite‐based devices is a crucial factor in assessing their potential for practical applications. Figure S49 (Supporting Information) illustrates the stability of 5% Ce^3+^ doped perovskite devices (PD) under conditions of high humidity, elevated temperature, open environmental exposure, and intense laser irradiation. Specifically, Figure S49a (Supporting Information) depicts the stability of the device when stored at room temperature (RT) with humidity levels maintained at 80 ± 5%. After a duration of 25 d, the device retained ≈97.6% of its initial response. In addition, following continuous exposure to a temperature of 80 °C for 25 d within a nitrogen‐filled glove box, the device's response was ≈93.4% of its initial value. (Figure S49c, Supporting Information). In addition, after continuous irradiation with 320 nm laser (127.3 µW cm^−2^) in ambient air (30 ± 5% relative humidity) at room temperature for 9 h, the response of PD was about 88% of the initial response (Figure S49d, Supporting Information). These results demonstrate that 5% Ce^3+^ doped PD exhibits excellent stabilities. For performance evaluation, there are more than 10 devices of each type. At the same time, the repeatability pf the PDs was evaluated. For each type of PD, at least 10 devices were systemically tested. For example, device performances of ten 5% Ce^3+^ PDs were statistically analyzed in Figure S50 (Supporting Information). The responsivity distribution of the devices is relatively dense, indicating that the devices have high repeatability.

## 3 Conclusion

In summary, we have demonstrated a universal strategy to dynamically modulate the PR of MHP ferroelectrics‐based heterojunctions via regulation of ferroelectric residual polarizations by applying different polarization voltage. The ferroelectric Ce^3+^ doped BDA_0.7_(BA_2_)_0.3_EA_2_Pb_3_Br_10_ films with strong UV absorption was selected as the model for UV polarized photodetection. By utilizing a tunable FPPE, the UV polarized photodetection with controllable PR was realized by varying the ferroelectric polarization voltages. The corresponding mechanism lies in the anisotropic interaction between the tunable built‐in electric field at the heterojunction and the polarized light, which can be proved by the polarization‐dependent dark *I–V*, *C–V*, and incidence angle dependent polarized photoresponses measurements. The variation trend of PR as a function of polarization voltage is consistent with the trend of *V*_oc_ and *V*_bi_. To validate the general applicability of this strategy, another three distinct types of 2D MHP ferroelectrics were used to fabricate heterojunction‐based polarized sensitive PDs. The polarization characteristics of the three PDs showed that ferroelectric polarization voltage can effectively modulate PR, regardless of material type, device structure, or polarized light wavelength. A proof‐of‐concept for encrypted UV optical communications using BDA_0.7_(BA_2_)_0.3_EA_2_Pb_3_Br_10_:Ce^3+^ films was demonstrated, highlighting improved anti‐interference capability. This approach provides an effective strategy for developing high‐performance, self‐powered polarized sensitive PDs using MHPs and enhances the understanding of FPPE in polarized sensitive photodetection, and provides a broad platform for fabricating polarized optoelectronic devices beyond MHPs.

## Conflict of Interest

The authors declare no conflict of interest.

## Supporting information



Supporting Information

## Data Availability

Research data are not shared.
