# Supplementary material for: 2D Perovskite Heterojunction‐Based Self‐Powered Polarized Photodetectors with Controllable Polarization Ratio Enabled by Ferro‐Pyro‐Phototronic Effect
Source: Adv Sci (Weinh). 2025 Jan 22;12(11):2414422. doi: 10.1002/advs.202414422 (PMC11923868; doi:10.1002/advs.202414422)
Supplement: Supplementary file 1 — Supporting Information [file ADVS-12-2414422-s001.docx]

Supporting Information

2D perovskite heterojunction-based self-powered polarized photodetectors with controllable polarization ratio enabled by ferro-pyro-phototronic effect

Xiaoran Yang^1^, Binyi Zhou^1^, Meitong Guo^1^, Yao Liu^1^, Ridong Cong^1^, Leipeng Li^1, 2^, Wenqiang Wu^3^, Shufang Wang^1^, Linjuan Guo^1, *^, Caofeng Pan^3, *^, Zheng Yang^1, 2, *^

**Experimental Section:**

*Materials.*

The SnO_2_ colloid solution oxide (15 wt% in H_2_O colloidal dispersion), N, N-Dimethylformamide (DMF, 99.9%), Dimethyl sulfoxide (DMSO, 99.9%), Cerium acetate (Ce(acac)_3_·xH_2_O, 99.9%), Polyethyleneimine (PEIE, 80% ethoxylated solution), hydriodic acid (HI, 57 wt%, 99.99%), ethylamine (EA, 68.0% - 72.0% in H_2_O), n-butylamine (BA, 99.5%), 1,4-Diaminobutane (BDA, 99%), 1-Methyl-2-pyrrolidinone (NMP, 99.5%), and Chlorobenzene (99.5%) were purchased from Shanghai Macklin Biochemical Co., Ltd. Lead bromine (PbBr_2_, 99.99%), Butylamine bromine (BABr, 99.99%), Butanediamine bromine (BDABr_2_, 99.99%), Ethylamine bromine (EABr, 99.99%), Lead iodide (PbI_2_, 99.99%), Methanaminium iodide (MAI, 99.99%), Methylamine acetate (MAAc, 99.99%), [6, 6]-Phenyl C61 butyric acid methyl ester (PC61BM), Poly(3-hexylthiophene-2, 5-diyl) (P3HT), Poly[bis(4-phenyl)(2, 4, 6-triMethylphenyl)aMine] (PTAA), and Poly(3, 4-ethylene dioxythiophene)-poly(styrene sulfonate) (PEDOT: PSS (4083)) were purchased from Xi'an Yuri Solar Co., Ltd. BDAI_2_, EAI, BAI, and TMIMI were prepared by reacting corresponding amines with HI. All the chemicals were used as received without further puriﬁcation. Indium tin oxide (ITO)/glass substrate (sheet resistance ≤ 8 Ω) were purchased from Advanced Election Technology Co., Ltd.

*Preparation of precursor solutions.*

Preparation of (BDA_0.7_(BA_2_)_0.3_)EA_2_Pb_3_Br_10_: x% Ce^3+^ (x = 0, 1, 3, 5, 7, and 10) precursor solutions: 0.3 M D-J or R-P 2D perovskite precursor solutions (the solution concentration is set by the Pb^2+^ concentration) were prepared by fully dissolving BDABr_2_ (or BABr), EABr, PbBr_2_ with the specific stoichiometric ratio in a mixed solvent containing DMF and DMSO (volume ratio = 4:1). Then, MAAc was added into the solution with a concentration of Ac^-^ to be 0.2 M. The BDA_0.7_(BA_2_)_0.3_EA_2_Pb_3_Br_10_ perovskite precursor solutions were prepared by mixing the pure DJ and pure RP perovskite solution with the volume ratio of 7:3. Finally, Ce(acac)_3_·xH_2_O with molar ratio of 1%, 3%, 5%, 7%, and 10% (the ratio is set by the concentration of Pb^2+^) were added into the mixed perovskite precursor solutions, respectively. Preparation of BDAEA_2_Pb_3_Br_10_ precursor solution: the 0.3 M D-J 2D perovskite precursor solution (the solution concentration is set by the Pb^2+^ concentration) was prepared by fully dissolving BDABr_2_, EABr, PbBr_2_ with the specific stoichiometric ratio in a mixed solvent containing DMF and DMSO (volume ratio = 4:1). Then, MAAc was added into the solution with a concentration of Ac^-^ to be 0.2 M. Preparation of (BDA_0.7_(BA_2_)_0.3_)MA_4_Pb_5_I_16_ precursor solution: the 0.4 M D-J or R-P 2D perovskite precursor solution (the solution concentration is set by the Pb^2+^ concentration) was prepared by fully dissolving BDAI_2_ (or BAI), MAI, PbI_2_ with the specific stoichiometric ratio in a mixed solvent containing DMF and DMSO (volume ratio = 9:1). Then, MAAc was added into the solution with a concentration of Ac^-^ to be 0.5 M. The BDA_0.7_(BA_2_)_0.3_MA_4_Pb_5_I_16_ perovskite precursor solutions were prepared by mixing the pure DJ and pure RP perovskite solution with the volume ratio of 7:3. Finally, TMIMI was added ito the mixed perovskite precursor solution with molar ratio of 0.05/1 (the ratio is set by the concentration of Pb^2+^). Preparation of BA_2_EA_2_Pb_3_I_10_ precursor solution: a mixture of 114.1 mg of PbI_2_, 33.2 mg of BAI, and 28.5 mg of EAI powder was dissolved in a solution containing DMF, DMSO, and NMP (volume ratio = 8:2:1). Then, MAAc was added into the solution with a concentration of Ac^-^ to be 0.33 M. All the precursor solutions were filtered with 0.22 μm PTFE filter membrane before use. A colloidal SnO_2_ aqueous dispersion with a concentration of 15 wt% was diluted to 5 wt% using deionized water and stirred at room temperature for two hours. The PC61BM solution was prepared by dispersed it with a concentration of 10 mg mL^-1^ in chlorobenzene. The P3HT solution was prepared by dispersed it with a concentration of 10 mg/mL in chlorobenzene. The PTAA and P3HT mixed solution was prepared by dispersed P3HT with a concentration of 1 mg/mL and PTAA with a concentration of 2 mg/mL in chlorobenzene.

*Fabrication of the heterojunction-based PDs.*

The entire process of device fabrication is carried out in ambient air (temperature ~ 23 ℃, RH ~ 30%-50%). Firstly, ITO substrates were ultrasonically washed in acetone, ethanol, and deionized water for 15 min successively, which were further treated with O_2_ plasma (70 W) for 30 min. Fabrication of devices with a ITO/(BDA_0.7_(BA_2_)_0.3_)EA_2_Pb_3_Br_10_: Ce^3+^/PTAA: P3HT/Au structure: the perovskite precursor solution was spin-coated onto ITO substrates at 4000 r.p.m. for 20 s with substrate temperature fixed at 70 ^o^C, followed by annealing at 80 ^o^C for 10 min. Then, PTAA: P3HT solution was deposited onto the perovskite films with a revolving speed of 1000 r.p.m., following by annealing at 100 ^o^C for 10 min. Finally, Au top electrode with a thickness of 50 nm was deposited by a thermal evaporator (0.1 Å/s) using a shadow mask. Fabrication of devices with ITO/BDAEA_2_Pb_3_Br_10_/PC61BM/Bi/Ag structure: the perovskite precursor solution was spin-coated onto ITO substrates at 4000 r.p.m. for 20 s with substrate temperature fixed at 70 ℃, followed by annealing at 80 ℃ for 10 min. Then, PC61BM solution was deposited onto the perovskite films with a revolving speed of 1000 r.p.m., following by annealing at 100 ^o^C for 10 min. Finally, 12 nm Bi and 80 nm Ag were successively deposited by a thermal evaporator (0.5 Å/s) using a shadow mask. Fabrication of devices with ITO/PEDOT: PSS/BDA_0.7_(BA_2_)_0.3_MA_4_Pb_5_I_16_/PC61BM/Bi/Ag structure: PEDOT: PSS (4083) was spin coated onto the ITO substrate at 5000 r.p.m. for 60 s, followed by annealing at 150 ^o^C for 10 minutes. Then the perovskite precursor solution was spin-coated at 3000 r.p.m. for 20 s with substrate temperature fixed at 70 ℃, followed by annealing at 80 ℃ for 10 min. Then, PC61BM solution was deposited onto the perovskite films with a revolving speed of 1000 r.p.m., following by annealing at 100 ^o^C for 10 min. Finally, top electrode of 12 nm Bi and 80 nm Ag top electrodes were successively deposited by a thermal evaporator (0.5 Å/s) using a shadow mask. Fabrication of devices with ITO/SnO_2_/BA_2_EA_2_Pb_3_I_10_/P3HT/MoO_3_/Ag structure: The dilute SnO_2_ solution (2.5 wt%) was spin coated onto the ITO substrate at 3000 r.p.m. for 30 s. Then the perovskite precursor solution was then spin-coated at 4000 r.p.m. for 20 s with substrate temperature fixed at 80 ℃, followed by annealing at 90 ℃ for 20 min. Then, PC61BM solution was deposited onto the perovskite films. Finally, 10 nm MoO_3_ and 100 nm Ag were successively deposited by a thermal evaporator (0.5 Å/s) using a shadow mask.

*Characterization of the materials.*

The XRD patterns were obtained by the Bruker D8 Advance X-ray diffractometer equipped with a Cu tube (λ = 1.5406 Å). UV-vis absorbance spectrum was measured using a Hitachi U4100 UV-Vis-NIR spectrophotometer at RT, with a wavelength range of 230 nm to 800 nm. XPS measurements were recorded using a photoelectron spectrometer (Thermo, ESCALAB 250 Xi) to analyze the working function of the perovskite films. Steady-state and time-resolved PL measurements were taken using an FLS920 (Edinburgh Instruments) fluorescence spectrometer with 360 nm excitation wavelength. The morphological analyses of the films and devices were examined by scanning electron microscopy (Nova NanoSEM450). GIWAXS measurements were performed using a small angle wide-angle X-ray scatterometer (Xeuss 2.0, Xenocs). Samples prepared on Si/SnO_2_ substrates used an X-ray beam (λ = 1.542 Å) at an incident angle of 0.20° for 30 s, and the scattered light was collected by the detector system at a distance of 150 mm vertically from the sample. Trap-state densities were determined using the SCLC method by conducting the dark *I-V* characteristics of the hole-only and electron-only devices. The structure is ITO/PEDOT: PSS/perovskite/P3HT/Au for hole-only devices. PEDOT: PSS (4083) solution was spin-coated at 5000 r.p.m. for 60 s in air and then annealed at 150 ℃ for 30 min. After the spin coating of the perovskite layer, the P3HT layer (10 mg mL^-1^ in chlorobenzene) was spin-coated at 2000 r.p.m. for 60 s. Finally, the 80 nm Au electrode was deposited using thermal evaporation through a shadow mask. The structure is ITO/SnO_2_/perovskite/PC61BM/Ag for electron-only devices. The dilute SnO_2_ solution (2.5 wt%) was spin-coated on the ITO at 3000 r.p.m. for 30 s and annealed at 150 ℃ for 30 min. After the spin coating of the perovskite layer, the PC61BM layer (10 mg mL^-1^ in chlorobenzene) was spin-coated at 2000 r.p.m. for 60 s. Finally, an 80 nm Au electrode was deposited using thermal evaporation through a shadow mask. The dark *I-V* curves were measured using the Keithley 2614b source meter.

*Photodetection and polarization characteristics measurements.*

The *I-V* characteristics and the *I-t* photoresponses of the PDs were recorded by coupling a Stanford SRS current preamplifier (SR570) and a Stanford SRS function generator (DS345). The temporal responses of the device were performed on a digital oscilloscope (Tektronix, MDO3104). The laser was periodically switched by transistor-transistor logic (TTL) potential to generate pulsed laser beams. The noise and *C-V* curves of the PDs were recorded by FS-Pro semiconductor parameter analyzer (Kailun Electronic Technology Co., Ltd.). The 261 nm, 320 nm, 360 nm, 405 nm, 532 nm, and 785 nm lasers (Changchun New Industries Optoelectronics Technology Co., Ltd.) were used as the light source. The light power intensity was calibrated using a silicon photodiode (PM100D). For wavelength-dependent photocurrent measurement, the monochromatic lights were provided by a mercury lamp equipped with a monochromator (NBeT, omno3001000). All devices were stored in the ambient atmosphere without any encapsulation during the measurements. The polarization direction of the linearly polarized laser is controlled by rotating the half-wave plate of a specific wavelength.

*Device parameters and testing details.*

1. The effective area of the device is 0.00785 cm^2^, defined as the illuminated area
2. All the on-off photoresonses of the devices are tested with a bias voltage of 0 V, except for the bias voltage-dependent pyroelectric response behaviors.
3. For polarized measurements, the linearly polarized lasers are reflected by a 45^o^ mirror onto the interface of top of the devices.
4. The response time is defined as the time required for the current to increase from 10% to 90% of the overall current, and the recovery time is the time required for the current to decrease from 90% to 10% of the overall current.
5. The carrier density of 5% Ce^3+^ doped perovskite films is 1.39×10^12^ cm^-3^, measured through the *C-V* measurements.

*The calculation formula used in the manuscript.*

1. The trap densities could be calculated according to the following equation:

$n_{\mathrm{trap}}=\frac{2\varepsilon_{0}\varepsilon_{r}V_{\mathrm{TFL}}}{eL^{2}}$ (1)

where *ε*_0_ is the vacuum permittivity, *ε*_r_ is the relative dielectric constant, *V*_TFL_ is the trap-filled limit voltage, *e* is the electron charge, and *L* is the film thickness. Here, *ε*_r_ was calculated according to equation:

$\varepsilon_{r}=C_{g}L/\varepsilon_{0}A$ (2)

Where C_g_ is geometric capacitance, L is the film thickness, ε_0_ is the vacuum permittivity, and A is the eﬀective area of devices.

1. The *R* and *D** were calculated following the equations below:

$R=\frac{\left( I_{\mathrm{photo}}-I_{d} \right)/S}{P}$ (3)

$D^{*}=\frac{({BS)}^{1/2}}{\mathrm{NEP}}=\frac{R{(BS)}^{1/2}}{{\bar{i_{n}^{2}}}^{1/2}}$ (4)

where *I*_photo_ is the photocurrent, *I*_d_ is the dark current, *S* is the active area, *P* is the light power density, *B* is the bandwidth, NEP is the noise equivalent power, ${\bar{i_{n}^{2}}}^{1/2}$is the root mean square value of the noise current.

Table S1. The PL lifetimes of the 0%, 1%, 3%, 5%, 7%, and 10% Ce^3+^ doped films.

| Lifetime (ns) | 0% Ce^3+^ | 1% Ce^3+^ | 3% Ce^3+^ | 5% Ce^3+^ | 7% Ce^3+^ | 10% Ce^3+^ |
| --- | --- | --- | --- | --- | --- | --- |
| τ_1_ | 0.49 | 1.05 | 0.98 | 1.75 | 0.89 | 0.97 |
| τ_2_ | 7.07 | 8.72 | 14.93 | 28.74 | 8.65 | 7.46 |

Table S2. The trap densities of the 0%, 1%, 3%, 5%, 7%, and 10% Ce^3+^ films.

| Trap density (×10^14^ cm^-3^) | 0% Ce^3+^ | 1% Ce^3+^ | 3% Ce^3+^ | 5% Ce^3+^ | 7% Ce^3+^ | 10% Ce^3+^ |
| --- | --- | --- | --- | --- | --- | --- |
| Electron | 5.04 | 5.36 | 4.26 | 3.27 | 4.68 | 4.82 |
| Hole | 6.67 | 7.55 | 6.45 | 5.86 | 6.20 | 7.17 |

**Figures.**


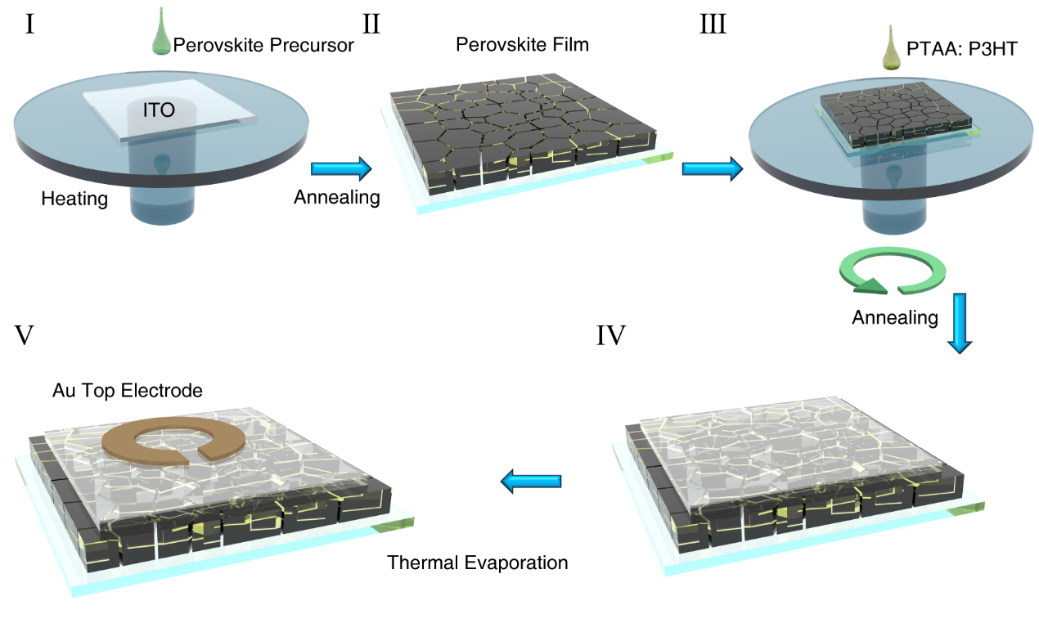


**Figure S1.** Schematic diagram showing the preparation process for (BDA_0.7_(BA_2_)_0.3_)(EA)_2_Pb_3_Br_10_: Ce^3+^ film-based polarized PDs.


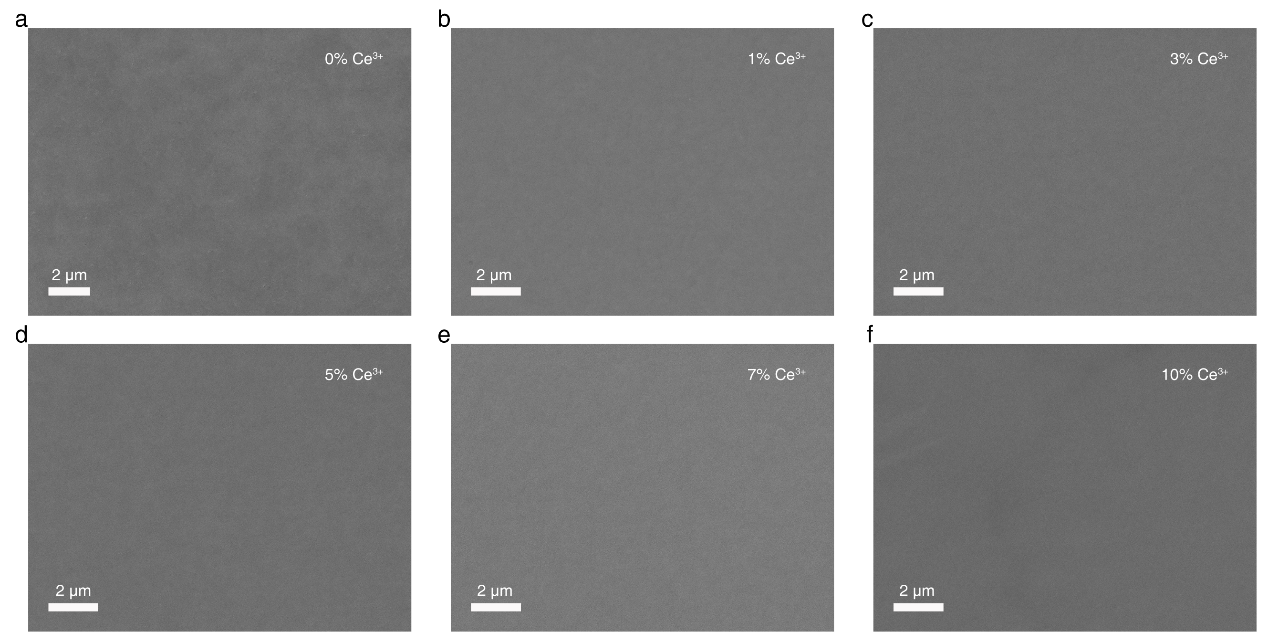


**Figure S2**. Top-view SEM images of the 0%, 1%, 3%, 5%, 7%, and 10% Ce^3+^ films.


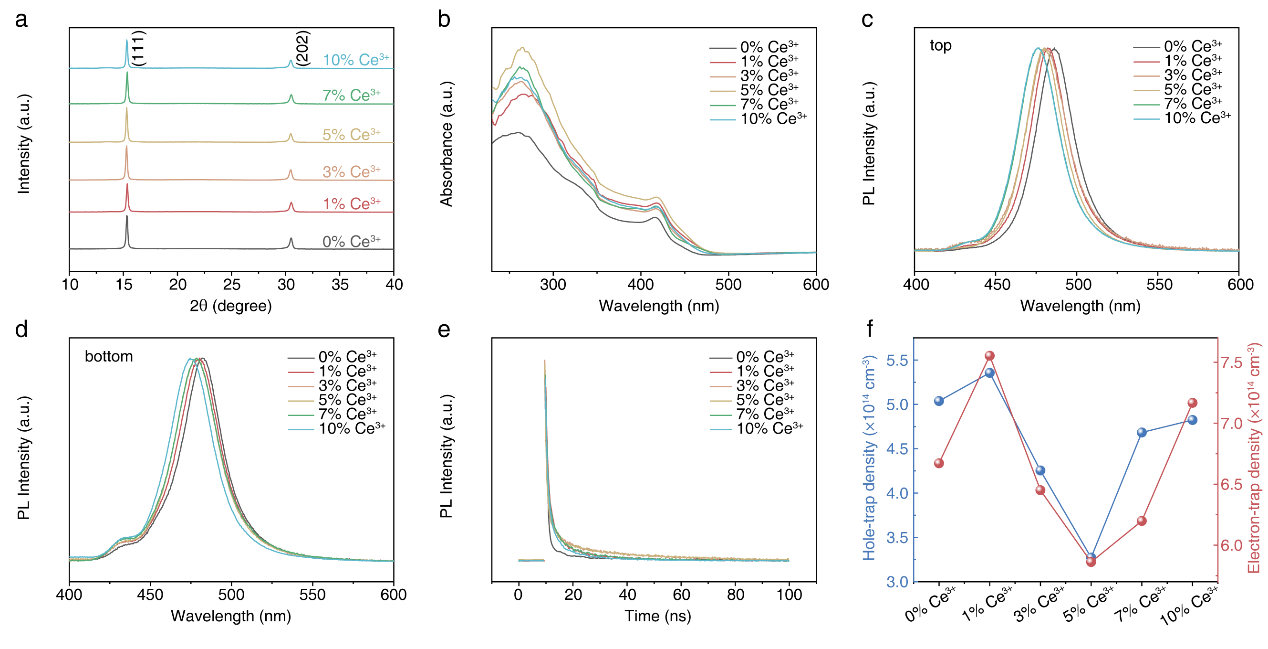


**Figure S3.** a) XRD, b) Abs, PL spectra excited from c) top side and d) bottom side, e) time-resolved PL decay curves, f) trap densities of hole and electron of the Ce^3+^ doped (BDA_0.7_(BA_2_)_0.3_)(EA)_2_Pb_3_Br_10_ films.


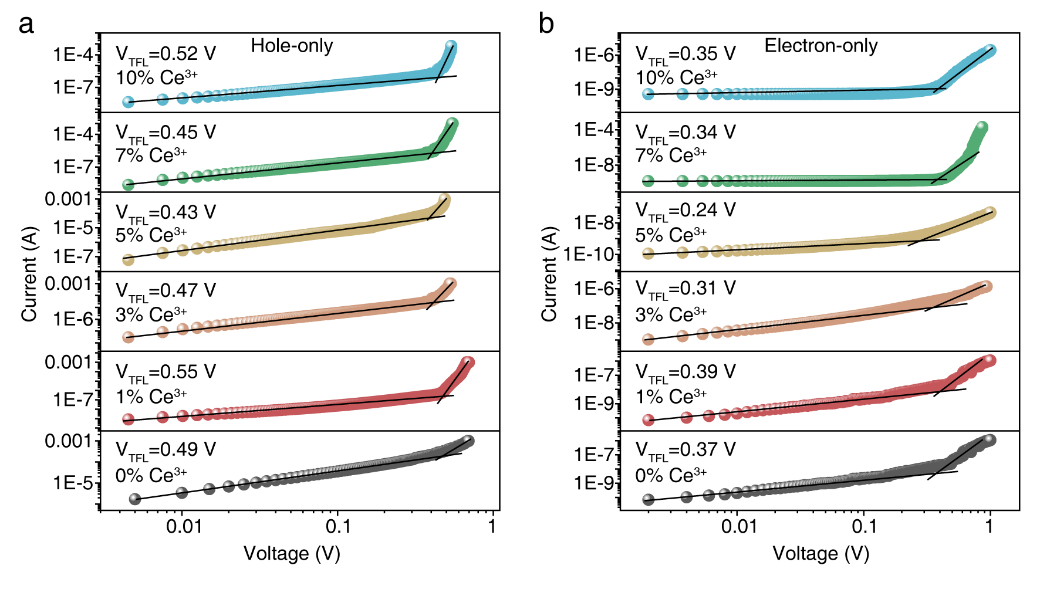


**Figure S4**. SCLC measurement results of the a) electron-only devices and b) hole-only devices based on the 0%, 1%, 3%, 5%, 7%, and 10% Ce^3+^ films.

**
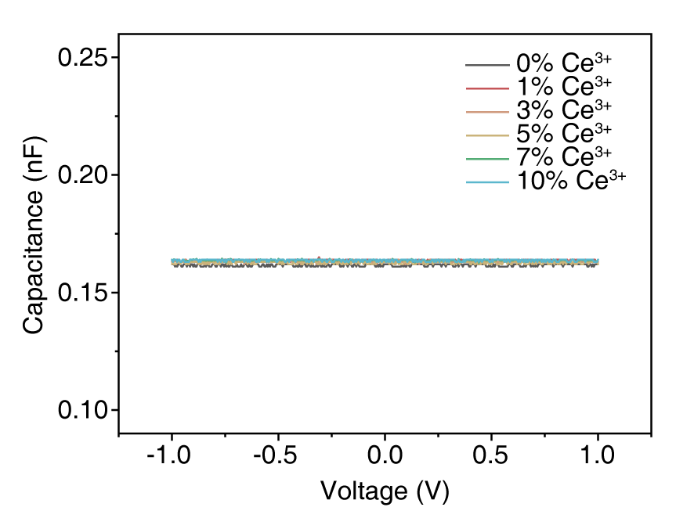
**

**Figure S5.** *C-V* characteristics of 0%, 1%, 3%, 5%, 7%, and 10% Ce^3+^ films.

**
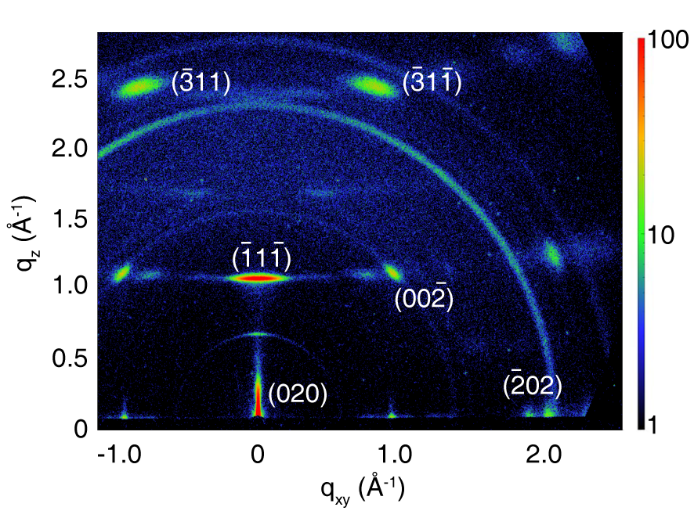
**

**Figure S6.** GIWAXS patterns of the (BDA_0.7_(BA_2_)_0.3_)(EA)_2_Pb_3_Br_10_: 5% Ce^3+^ film.


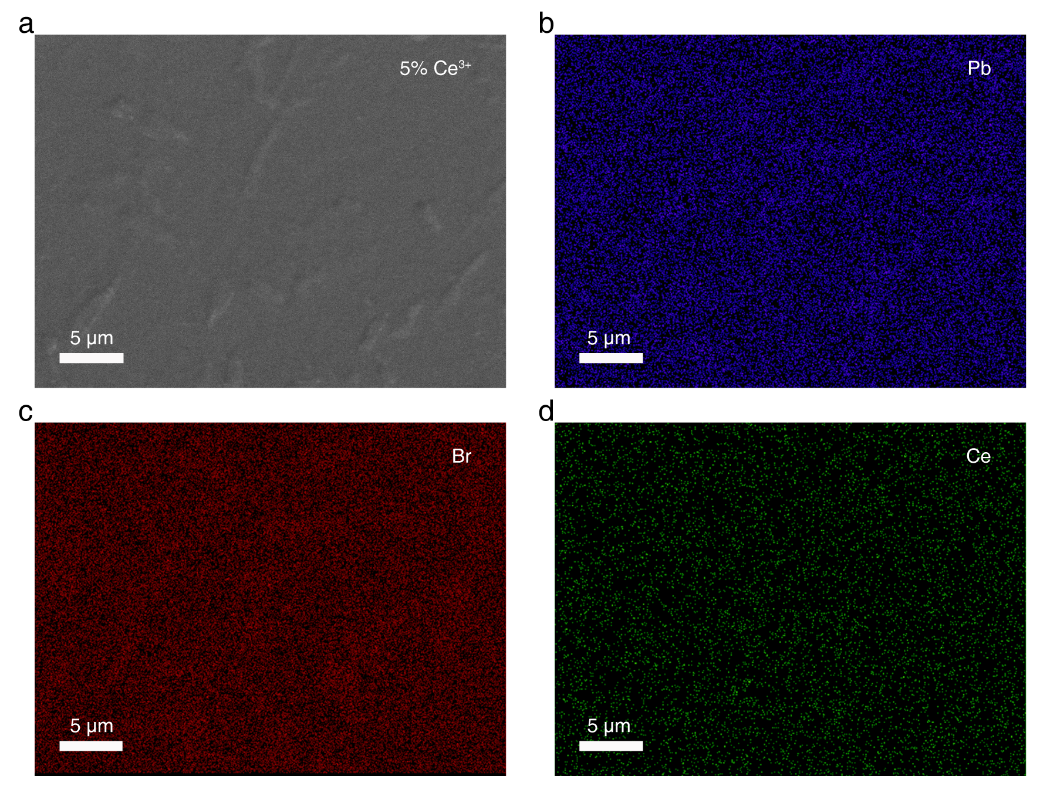


**Figure S7.** EDS mapping of the three detected elements b) Pb, c) Br, and d) Ce in (BDA_0.7_(BA_2_)_0.3_)(EA)_2_Pb_3_Br_10_: 5% Ce^3+^ film.


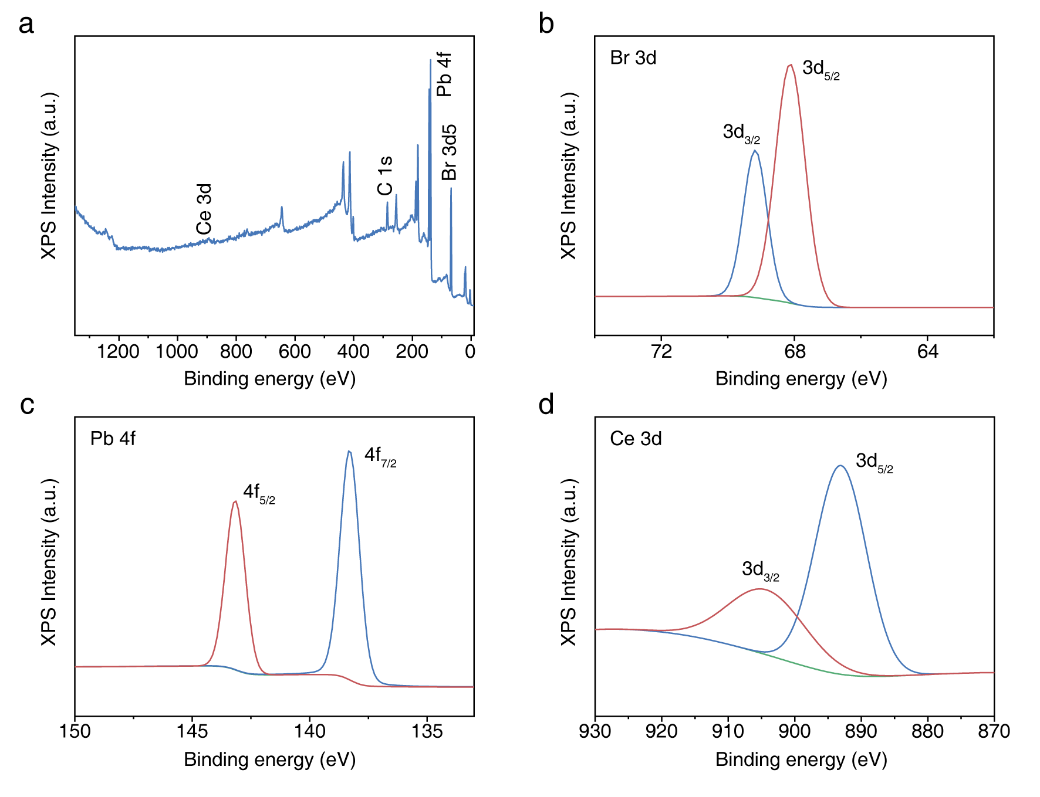


**Figure S8.** a) Full XPS spectrum of the (BDA_0.7_(BA_2_)_0.3_)(EA)_2_Pb_3_Br_10_: 5% Ce^3+^ film. XPS peaks of b) Br 3d, c) Pb 4f, d) Ce 3d derived from a).


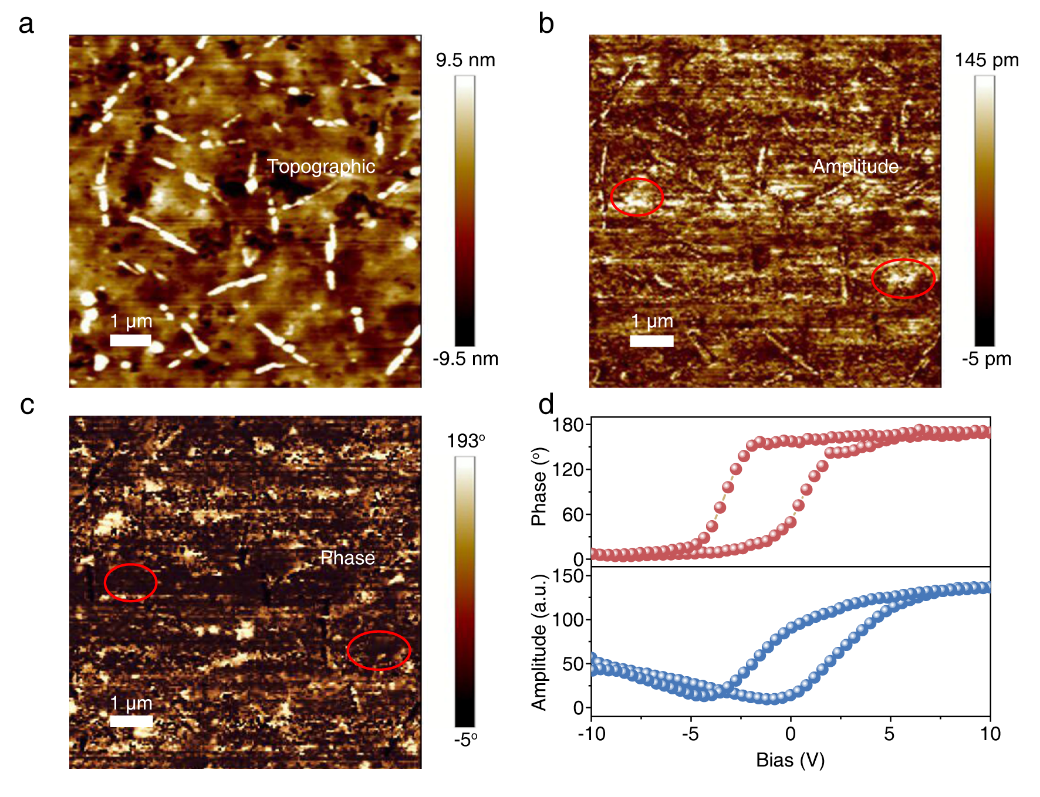


**Figure S9.** a) Topographic image, b) amplitude image, and c) PFM phase image of the (BDA_0.7_(BA_2_)_0.3_)(EA)_2_Pb_3_Br_10_: 5% Ce^3+^ film. d) Out-of-plane PFM hysteresis loops measured in the film surface, showing a hysteresis loop and a butterfly curve.

**
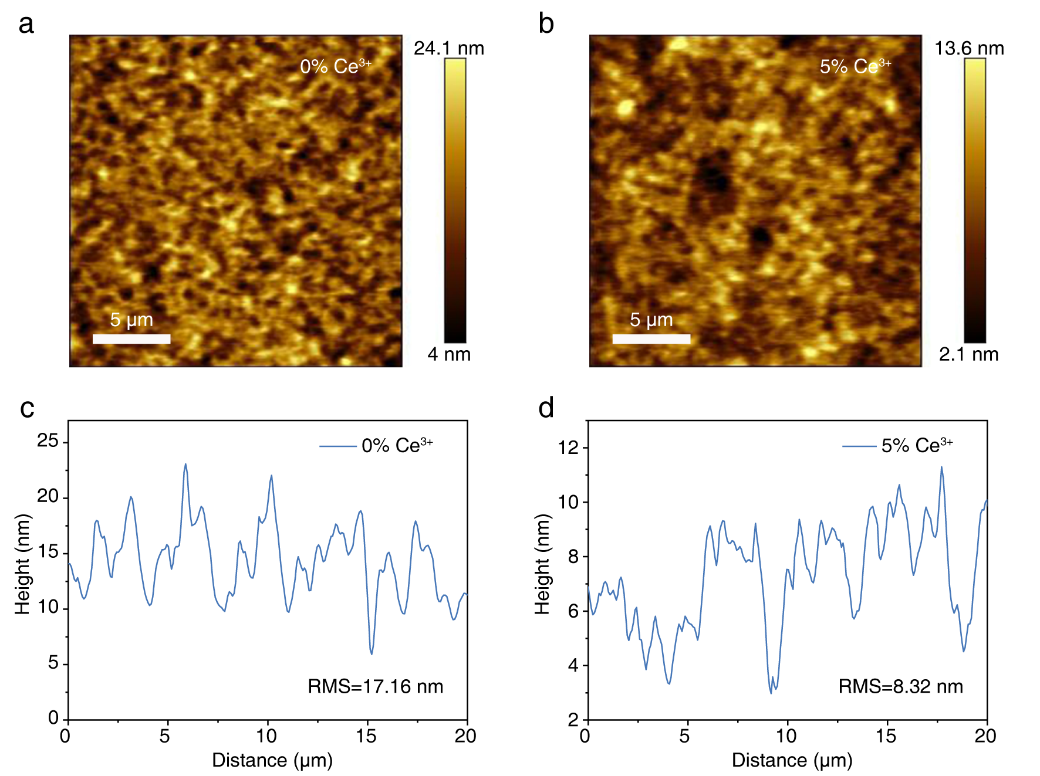
**

**Figure S10.** AFM images on the surface of a) 0% Ce^3+^ and b) 5% Ce^3+^ films. The roughness of a) 0% Ce^3+^ and b) 5% Ce^3+^ films derived from a) and b), respectively.

**
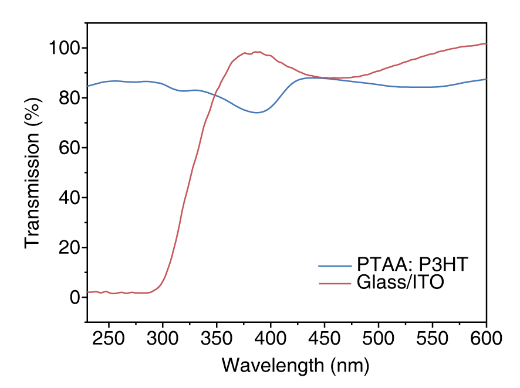
**

**Figure S11.** Transmission spectra of PTAA: P3HT film and glass/ITO substrate.


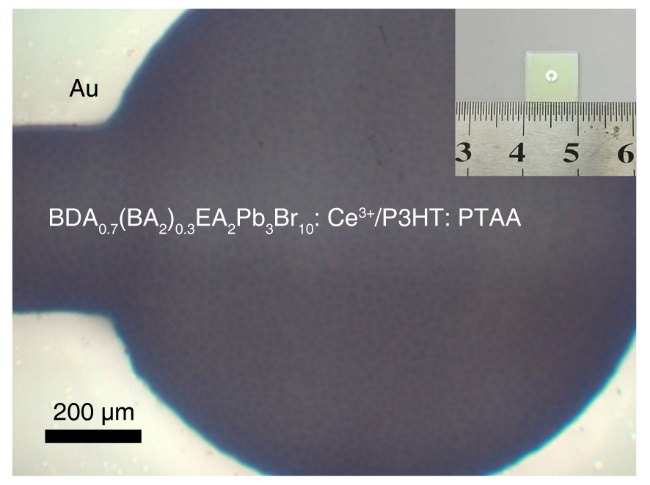


**Figure S12.** Optical microscope image of the device. The inset is a picture of the device.


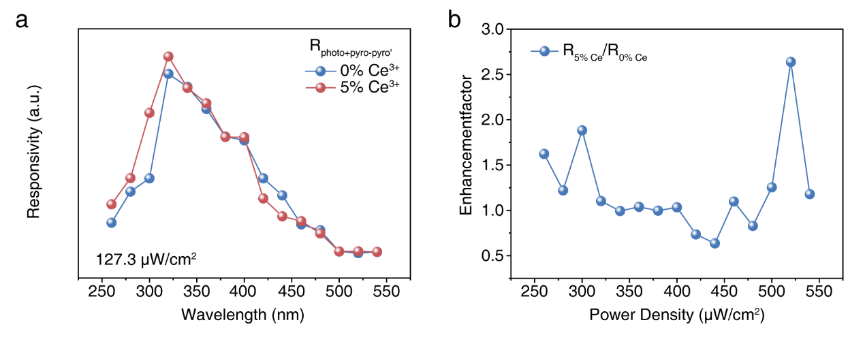


**Figure S13.** a) Wavelength-dependent responsivity (*R_photo+pyro-pyro'_*) of the PDs under zero bias based on 0% Ce^3+^ and 5% Ce^3+^ films. b) Enhancement factor for R_5% Ce_/R_0% Ce_, showing the enhanced UV photoresponses after Ce^3+^ doping.


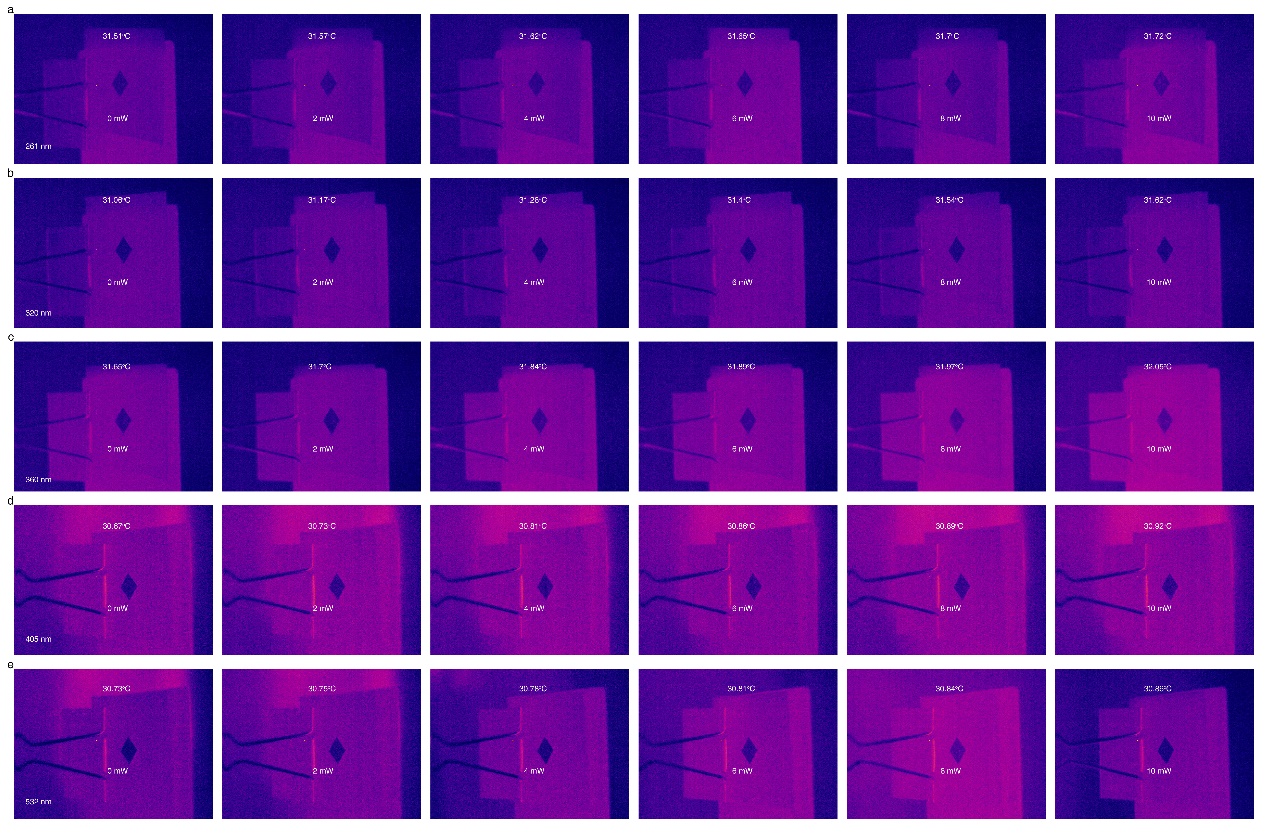


**Figure S14.** Thermograms of the 5% Ce^3+^ film illuminated with 261, 320, 360, 405, and 532 nm laser.


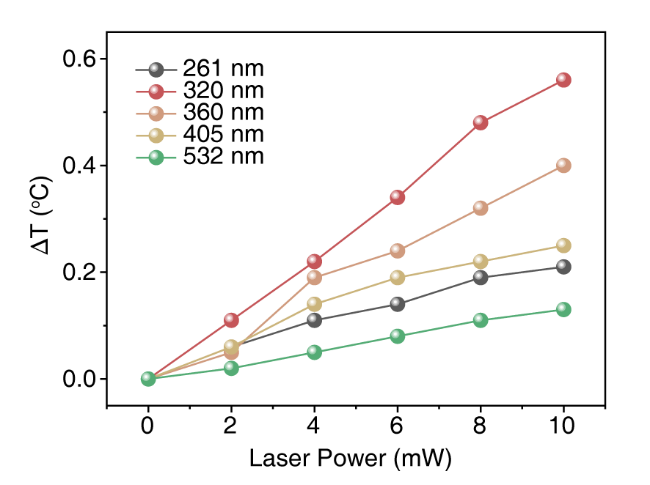


**Figure S15**. The temperature of the 5% Ce^3+^ film is illuminated with different lasers with different output power.


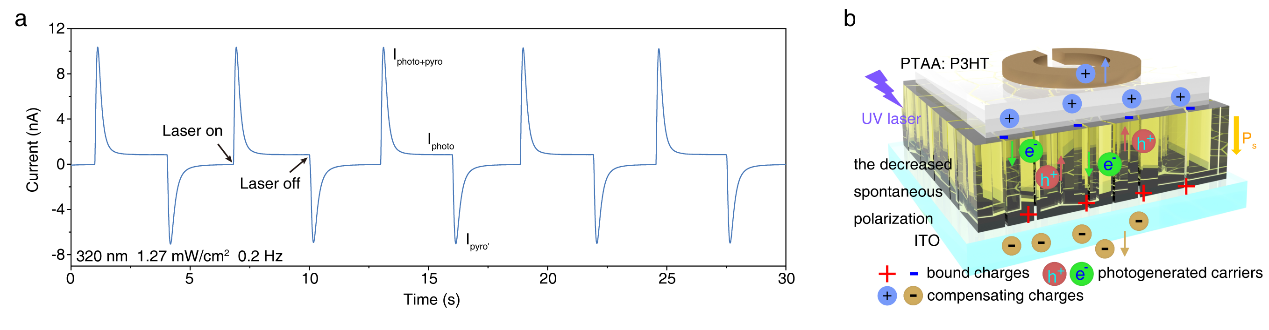


**Figure S16.** a) The on- off I-t curves of 5% Ce^3+^ self-powered PDs under 320 nm showing a typical four-stage response behavior. b) The schematic diagram of the fundamental working mechanism of ferro-pyro-phototronic effect-based self-powered PDs.


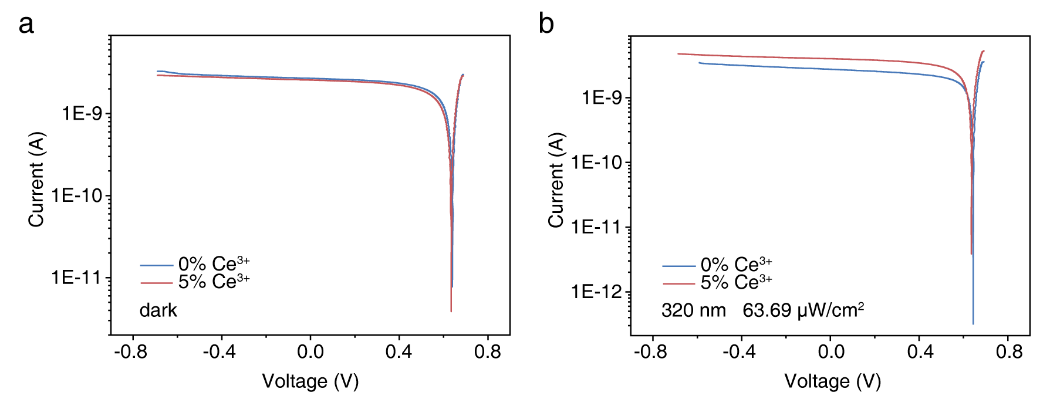


**Figure S17.** *I-V* curves of 0% and 5% Ce^3+^ film-based PDs under a) dark and b) 320 nm laser illumination with a power density of 63.69 μW cm^-2^.


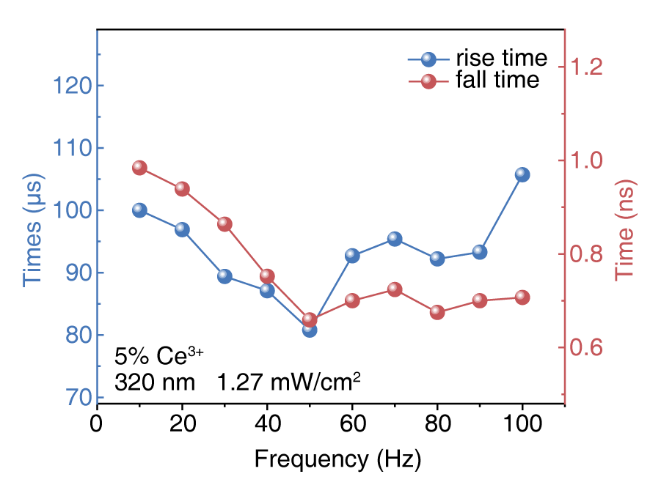


**Figure S18.** Frequency-dependent response times of (BDA_0.7_(BA_2_)_0.3_)(EA)_2_Pb_3_Br_10_: 5% Ce^3+^ film-based PD.


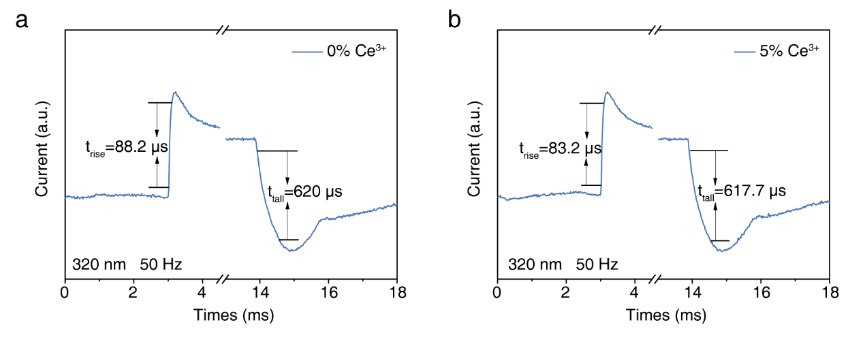


**Figure S19.** Temporal responses of (BDA_0.7_(BA_2_)_0.3_)(EA)_2_Pb_3_Br_10_ film-based PDs with a) 0% and b) 5% Ce^3+^.


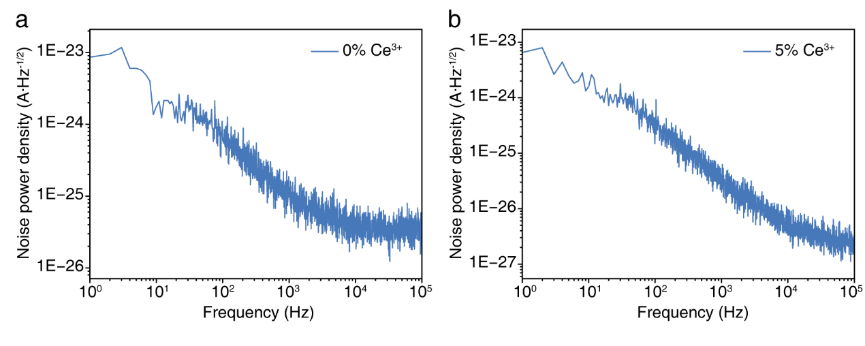


**Figure S20.** Noise current of (BDA_0.7_(BA_2_)_0.3_)(EA)_2_Pb_3_Br_10_ film-based PDs with a) 0% and b) 5% Ce^3+^.


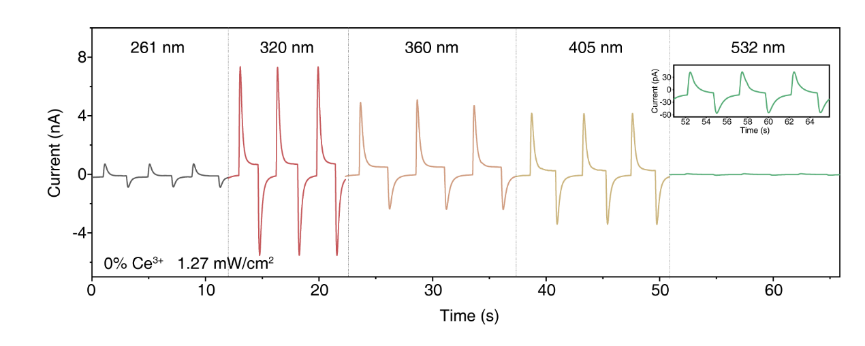


**Figure S21.** Spectral photoresponse behaviors of the 0% Ce^3+^ film-based PD under 261 nm, 320 nm, 360 nm, 405 nm, and 532 nm laser illuminations under zero bias.


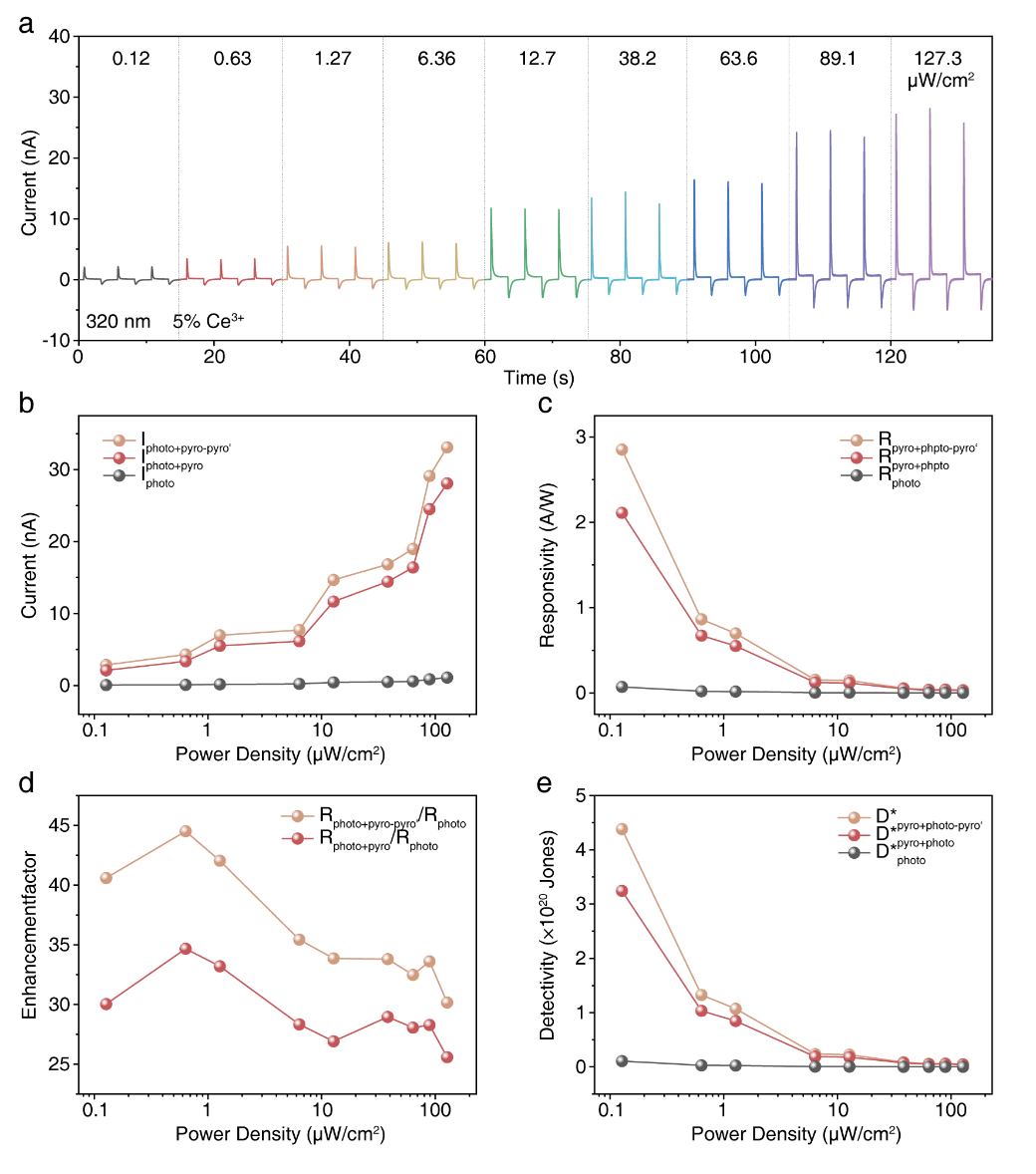


**Figure S22.** Self-powered performance of the ferro-pyro-phototronic effect enhanced (BDA_0.7_(BA_2_)_0.3_)(EA)_2_Pb_3_Br_10_: 5% Ce^3+^ film-based PD towards 320 nm laser. a) Photoresponses of the PD with different power densities from 0.12 to 127.3 μW cm^-2^. b) Three output currents extracted from a) the corresponding responsivities, c) and corresponding detectivities, e) of the PD as a function of the power density. d) Enhancement factor for R_pyro+photo-pyro'_/R_photo_ and R_pyro+photo_/R_photo_.


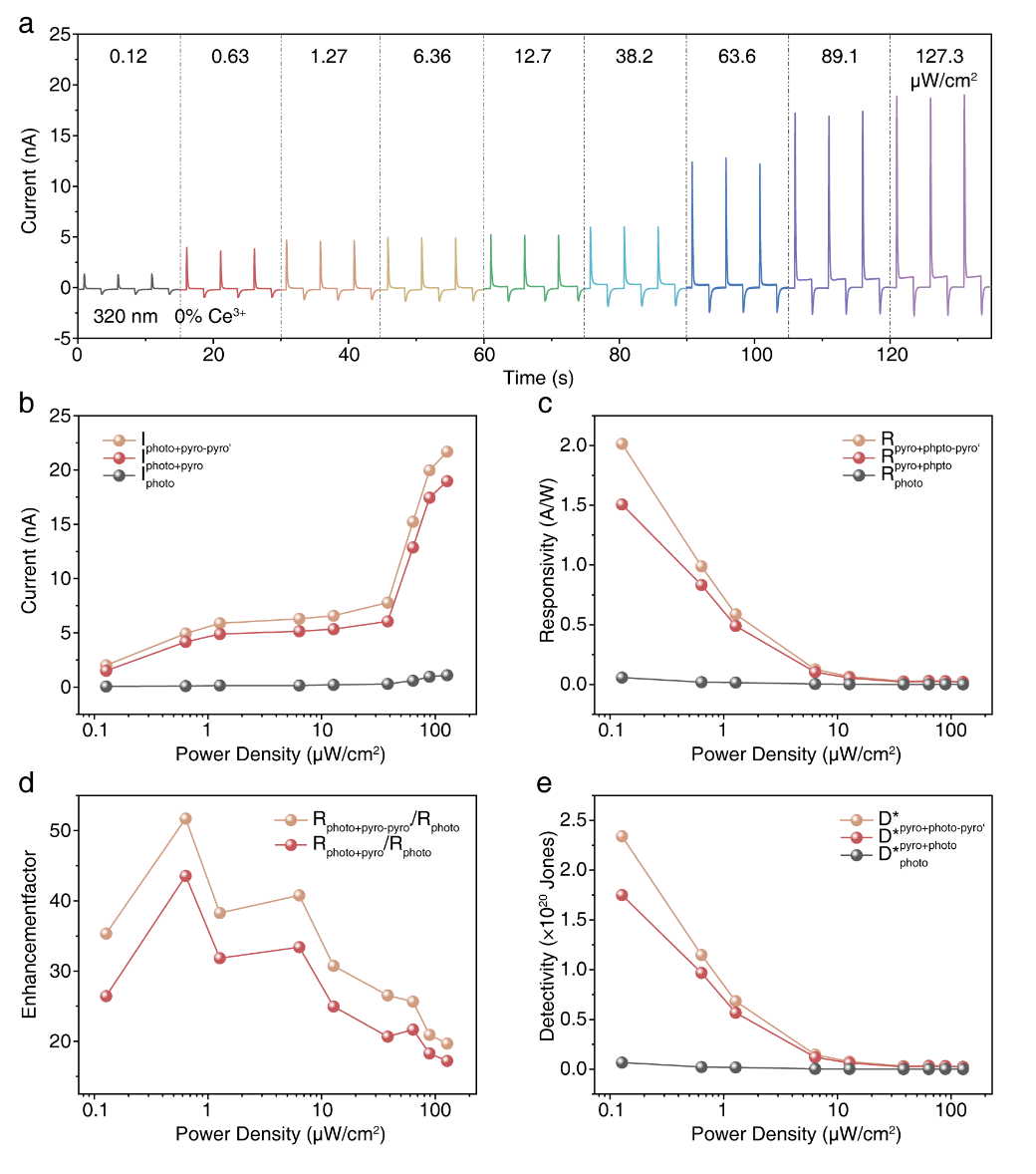


**Figure S23.** Self-powered performance of the ferro-pyro-phototronic effect enhanced (BDA_0.7_(BA_2_)_0.3_)(EA)_2_Pb_3_Br_10_ film-based PD towards 320 nm laser. a) Photoresponses of the PD with different power densities from 0.12 to 127.3 μW cm^-2^. b) Three output currents extracted from a) the corresponding responsivities, c) and corresponding detectivities, e) of the PD as a function of the power density. d) Enhancement factor for R_pyro+photo-pyro'_/R_photo_ and R_pyro+photo_/R_photo_.


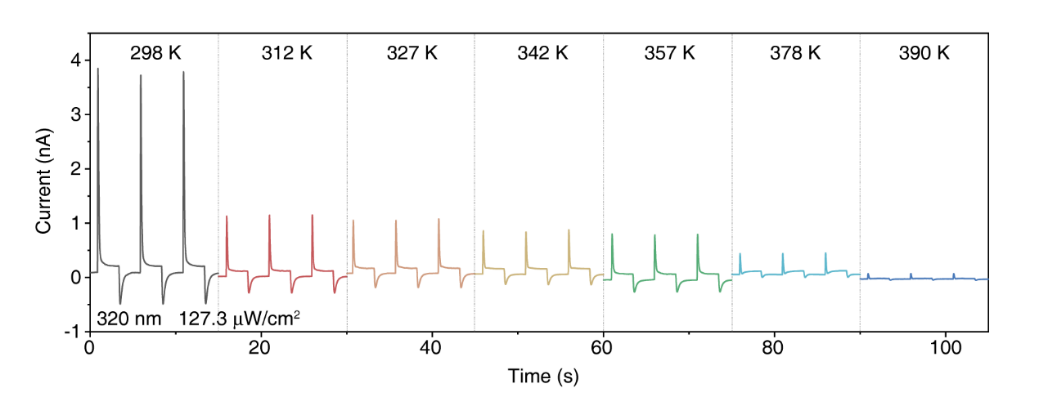


**Figure S24.** Temperature dependence of the photoresponses of the 5% Ce^3+^ film-based PD.


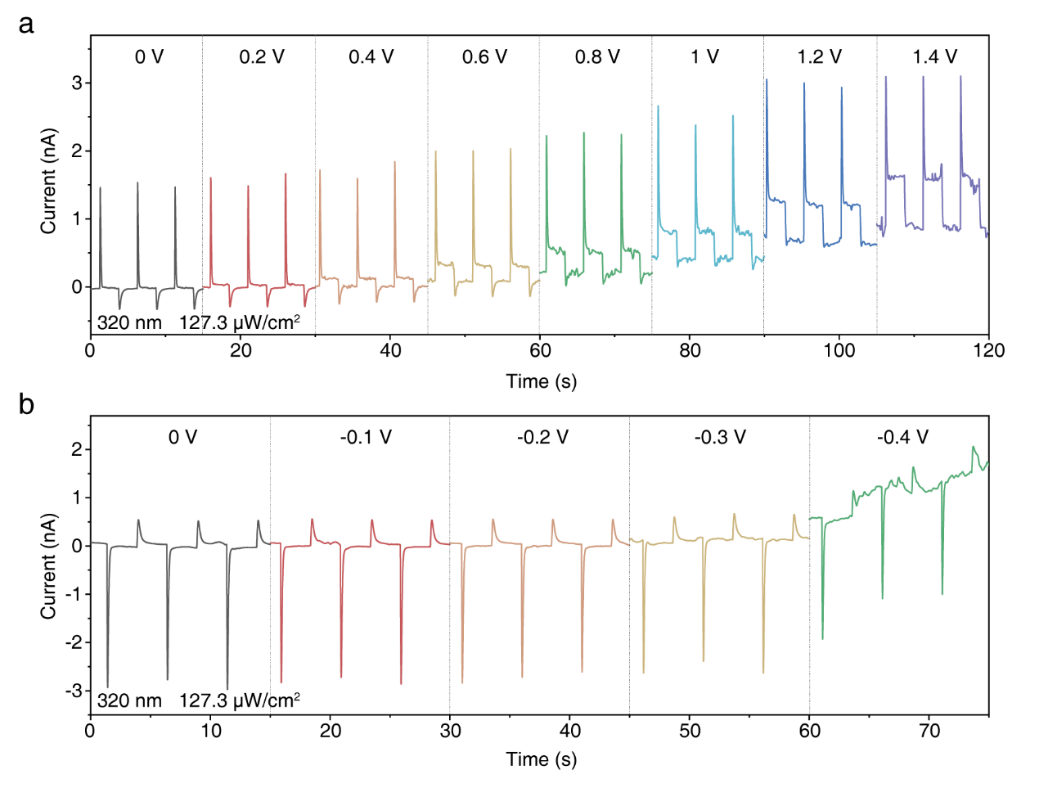


**Figure S25.** *I*-*t* characteristics of the 5% Ce^3+^ film-based PD at different a) forward bias from 0 to 1.4 V and b) reversed bias from 0 to -0.4 V under 320 nm laser illumination (127.3 μW cm^-2^).


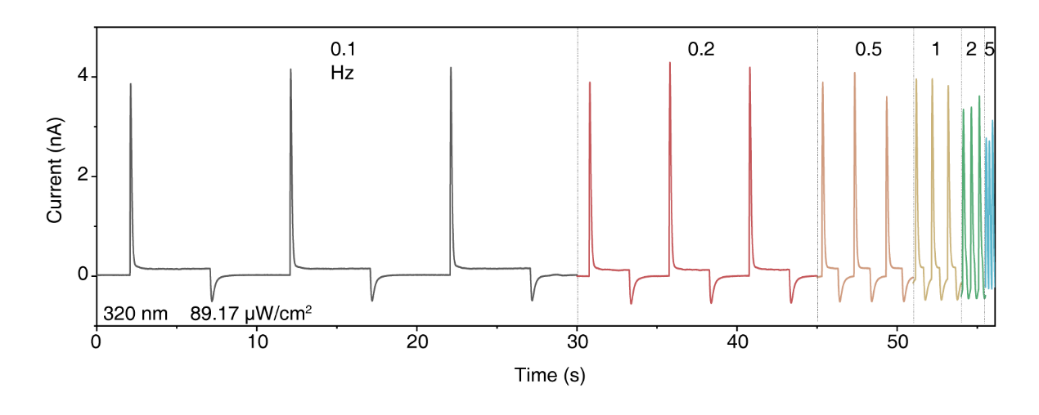


**Figure S26.** *I-t* response of 320 nm, 89.17 μW cm^2^ laser at different frequencies.


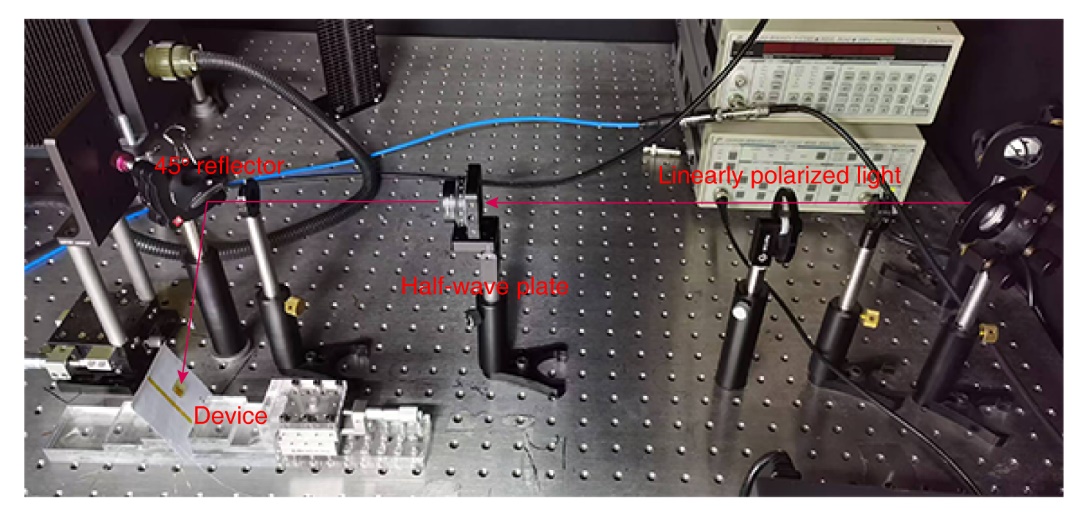


**Figure S27.** The photograph of the polarization characteristic measurement optical path.


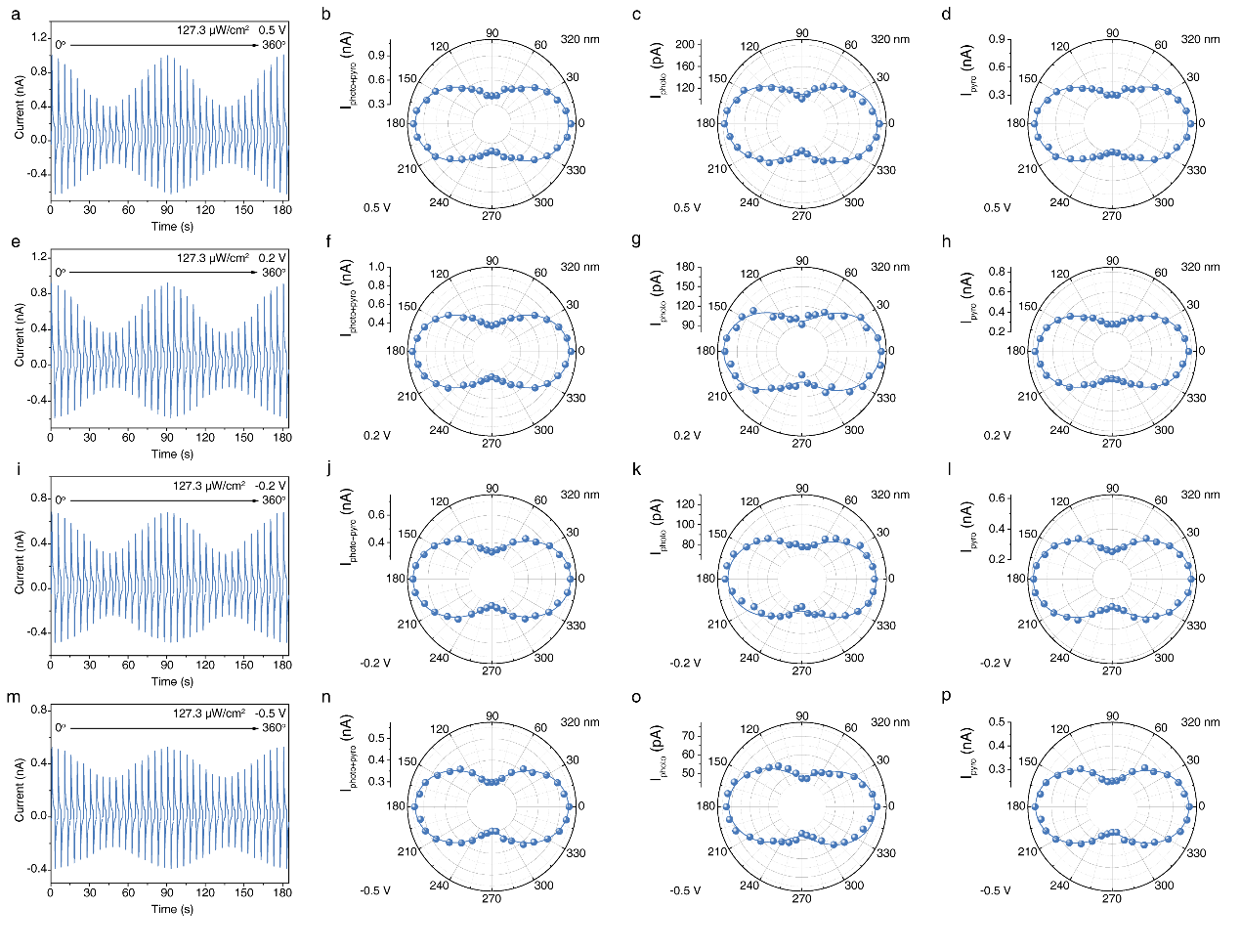


**Figure S28.** Photoresponses of the (BDA_0.7_(BA_2_)_0.3_)(EA)_2_Pb_3_Br_10_: 5% Ce^3+^ film-based PD under 320 nm laser with diﬀerent polarization angles at zero bias voltage after been applying with a) 0.5 V, e) 0.2 V, i) -0.2 V, and m) -0.5 V ferroelectric polarization voltage. Polar diagrams of the polarization-sensitive I_photo+pyro_, I_photo_ and I_pyro_ at zero bias for b-d) 0.5 V, f-h) 0.2 V, j-l) -0.2 V, and n-p) -0.5 V ferroelectric polarization voltage conditions.


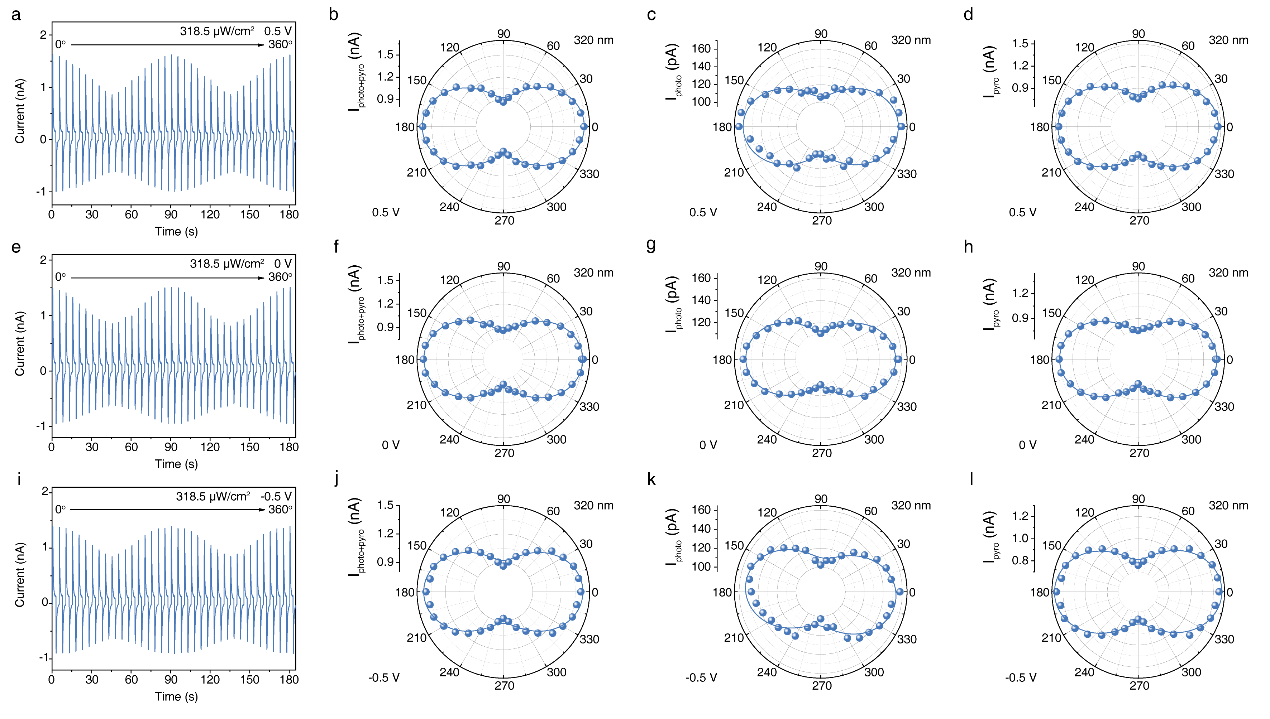


**Figure S29.** Photoresponses of the (BDA_0.7_(BA_2_)_0.3_)(EA)_2_Pb_3_Br_10_: 5% Ce^3+^ film-based PD under 320 nm laser with diﬀerent polarization angles at zero bias voltage after been applying with a) 0.5 V, e) 0 V, and i) -0.5 V ferroelectric polarization voltage. Polar diagrams of the polarization-sensitive I_photo+pyro_, I_photo_ and I_pyro_ at zero bias for b-d) 0.5 V, f-h) 0 V, and j-l) -0.5 V ferroelectric polarization voltage conditions.


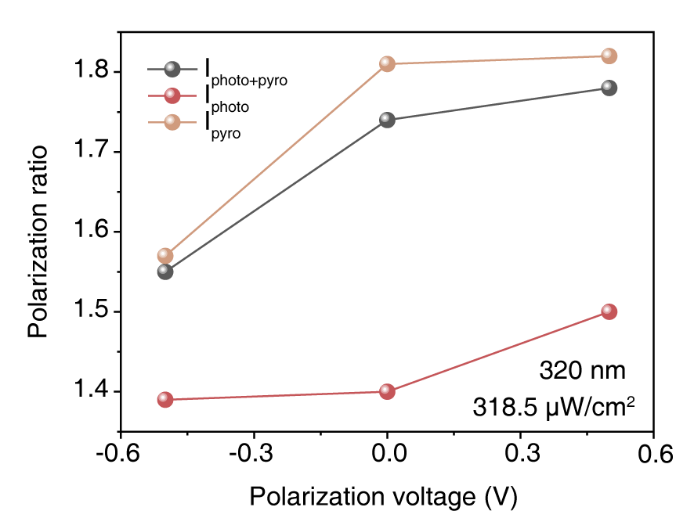


**Figure S30.** Polarization voltage-dependent PR of I_photo+pyro_, I_photo_, and I_pyro_ for (BDA_0.7_(BA_2_)_0.3_)(EA)_2_Pb_3_Br_10_: 5% Ce^3+^ film-based PD at light wavelength of 320 nm, with a power density of 318.5 μW cm^-2^.


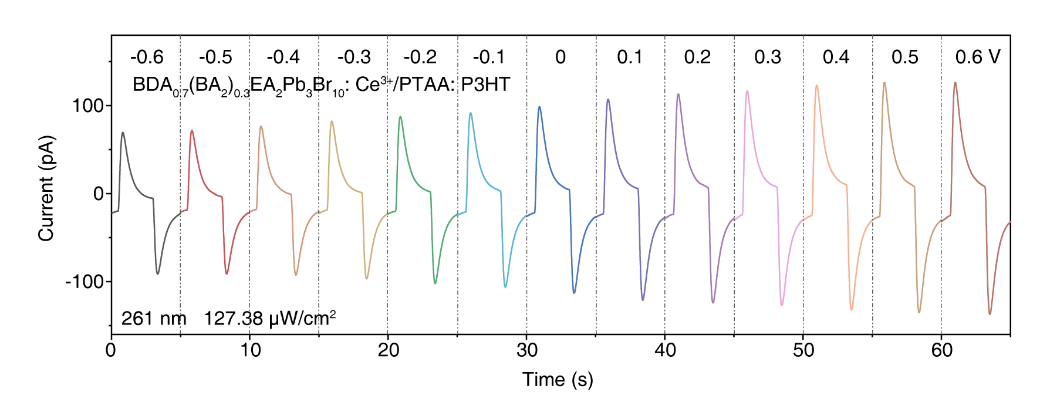


**Figure S31.** The zero bias photoresponses of the (BDA_0.7_(BA_2_)_0.3_)(EA)_2_Pb_3_Br_10_: 5% Ce^3+^ film-based PD towards 261 nm laser after been applying with different ferroelectric polarization voltages.


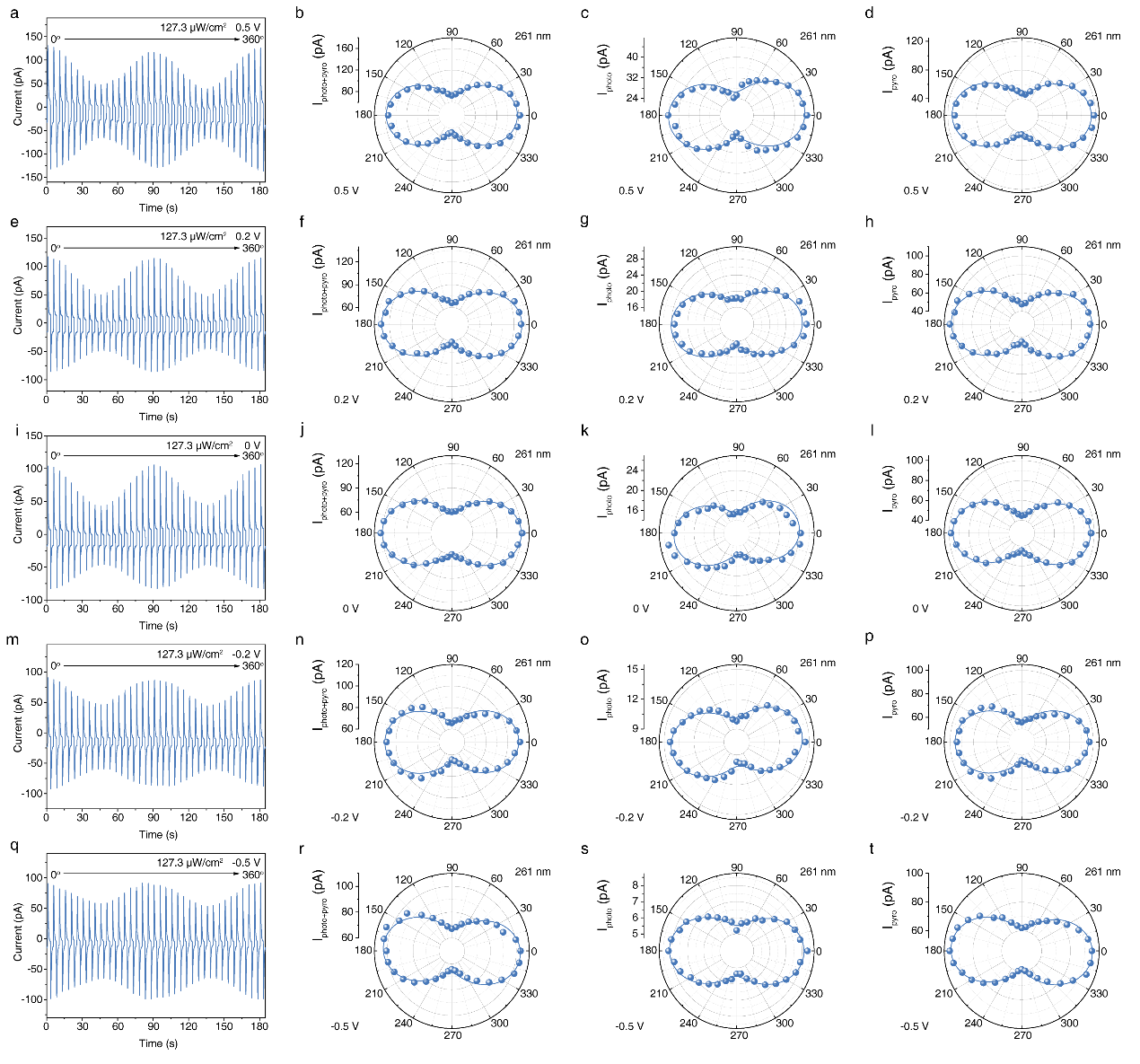


**Figure S32.** Photoresponses of the (BDA_0.7_(BA_2_)_0.3_)(EA)_2_Pb_3_Br_10_: 5% Ce^3+^ film-based PD under 261 nm laser with diﬀerent polarization angles at zero bias voltage after been applying with a) 0.5 V, e) 0.2 V, i) 0 V, m) -0.2 V, and q) -0.5 V ferroelectric polarization voltage. Polar diagrams of the polarization-sensitive I_photo+pyro_, I_photo_ and I_pyro_ at zero bias for b-d) 0.5 V, f-h) 0.2 V, j-l) 0 V, n-p) -0.2 V, and r-t) -0.5 V ferroelectric polarization voltage conditions.


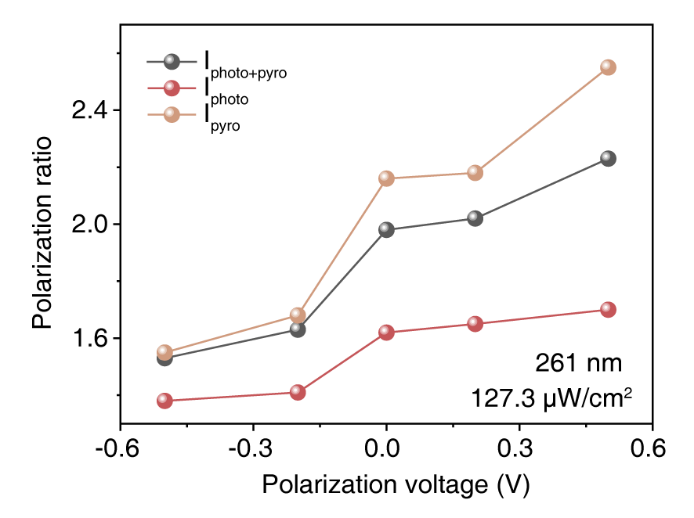


**Figure S33.** Polarization voltage-dependent PR of I_photo+pyro_, I_photo_, and I_pyro_ for (BDA_0.7_(BA_2_)_0.3_)(EA)_2_Pb_3_Br_10_: 5% Ce^3+^ film-based PD at light wavelength of 261 nm, with a power density of 127.3 μW cm^-2^.


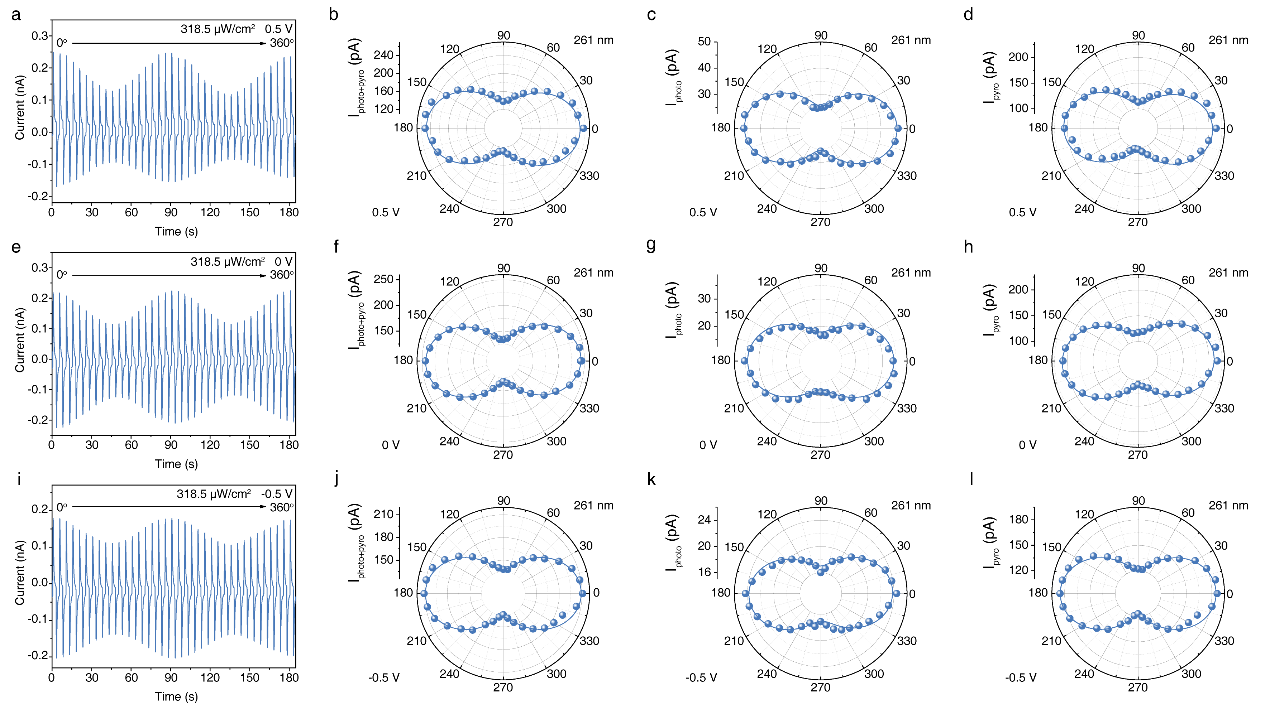


**Figure S34.** Photoresponses of the (BDA_0.7_(BA_2_)_0.3_)(EA)_2_Pb_3_Br_10_: 5% Ce^3+^ film-based PD under 261 nm laser with diﬀerent polarization angles at zero bias voltage after been applying with a) 0.5 V, d) 0 V, and g) -0.5 V ferroelectric polarization voltage. Polar diagrams of the polarization-sensitive I_photo+pyro_, I_photo_ and I_pyro_ at zero bias for b-d) 0.5 V, f-h) 0 V, and j-l) -0.5 V ferroelectric polarization voltage conditions.


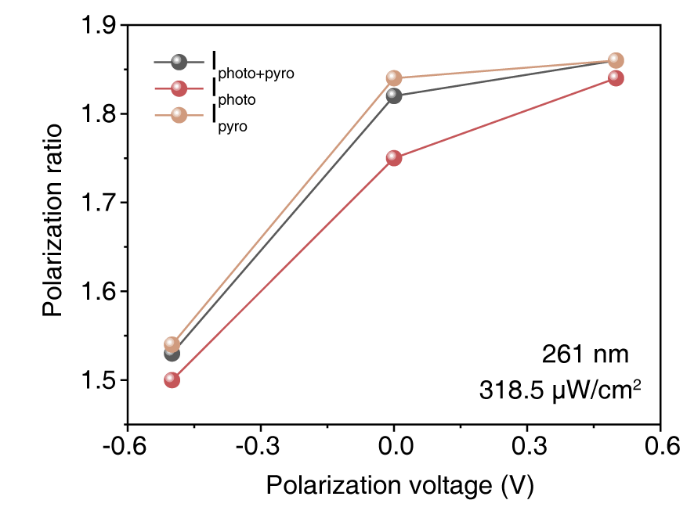


**Figure S35.** Polarization voltage-dependent PR of I_photo+pyro_, I_photo_, and I_pyro_ for (BDA_0.7_(BA_2_)_0.3_)(EA)_2_Pb_3_Br_10_: 5% Ce^3+^ film-based PD at light wavelength of 261 nm, with a power density of 318.5 μW cm^-2^.


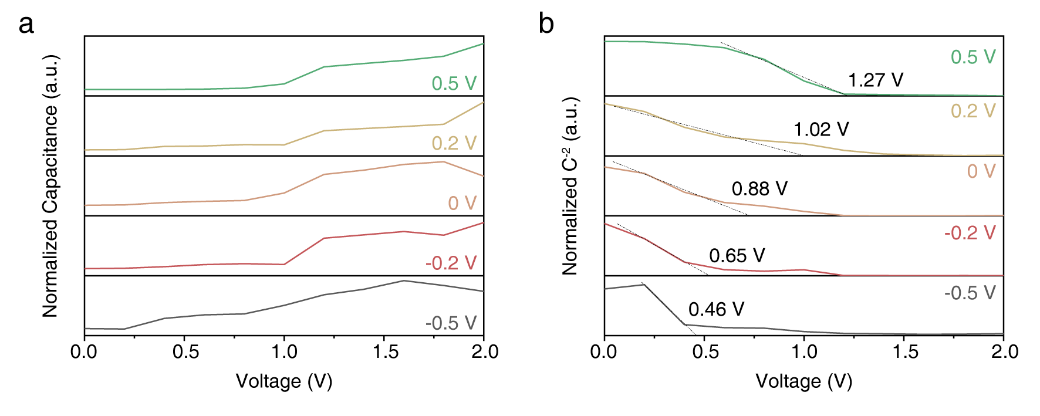


**Figure S36.** a) *C-V* curves of the (BDA_0.7_(BA_2_)_0.3_)(EA)_2_Pb_3_Br_10_: 5% Ce^3+^ film-based PDs after been applying with five different ferroelectric polarization voltages. b) Mott-Schottky curves derived from a), showing the V_bi_.

**
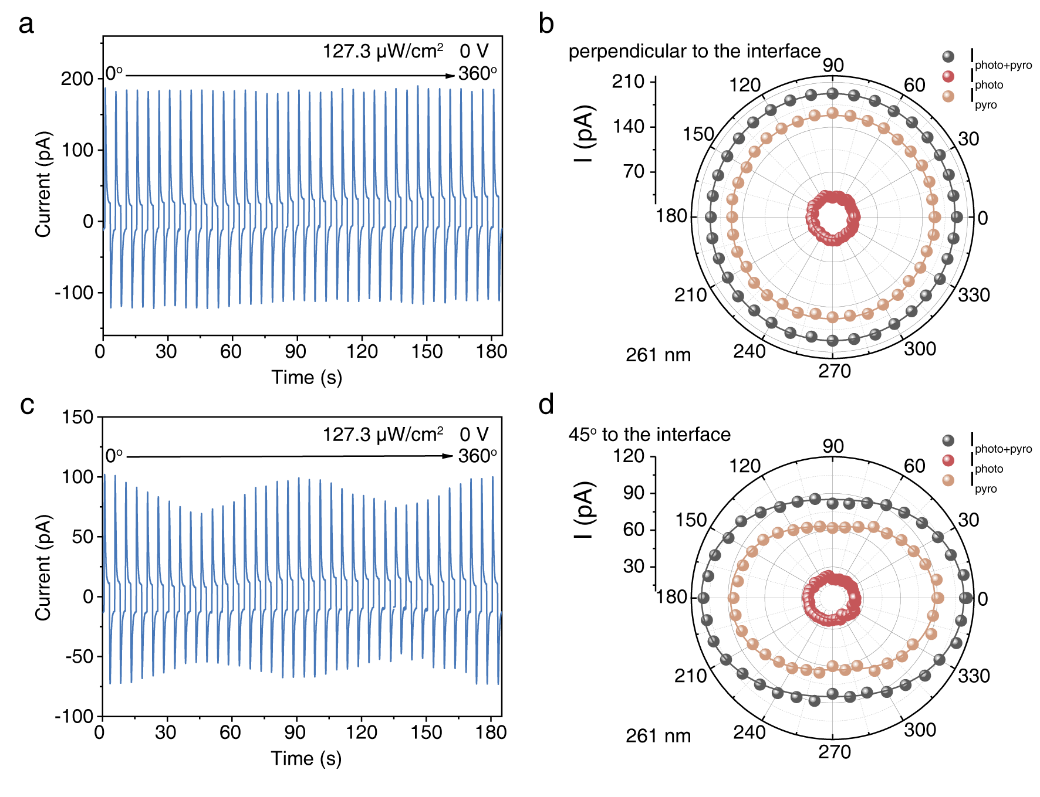
**

**Figure S37.** Photoresponses of the (BDA_0.7_(BA_2_)_0.3_)(EA)_2_Pb_3_Br_10_: 5% Ce^3+^ film-based PDs with diﬀerent polarization angles at zero bias voltage under 261 nm laser with incident angle of a) 0° and c) 45°. Polar diagrams of the polarization-sensitive I_photo+pyro_ I_photo_ and I_pyro_ at zero bias for b-c) b) 0° and d) 45° incident angle.

**
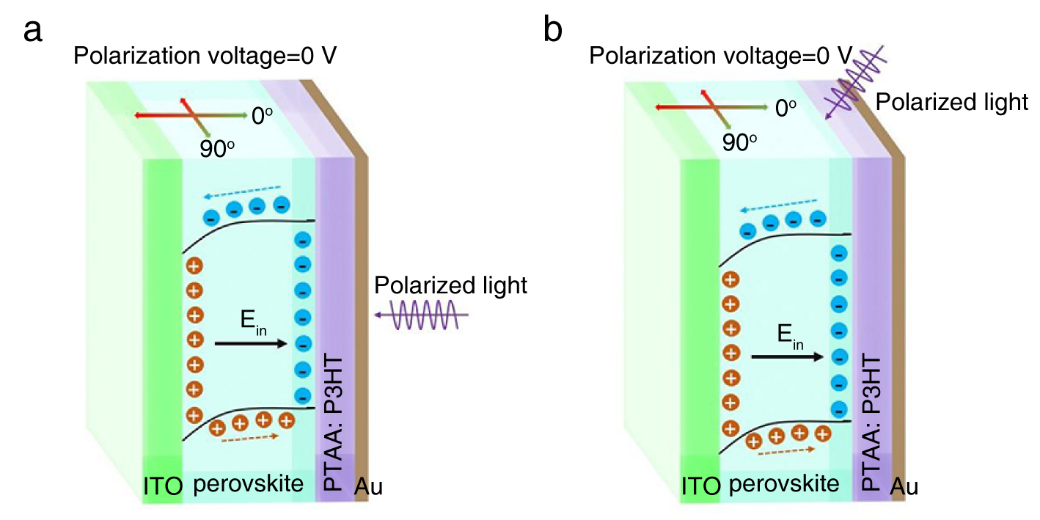
**

**Figure S38.** Energy band diagram of the (BDA_0.7_(BA_2_)_0.3_)(EA)_2_Pb_3_Br_10_: 5% Ce^3+^ film-based PD with polarized light incident angle at a) 0° and b) 45°.


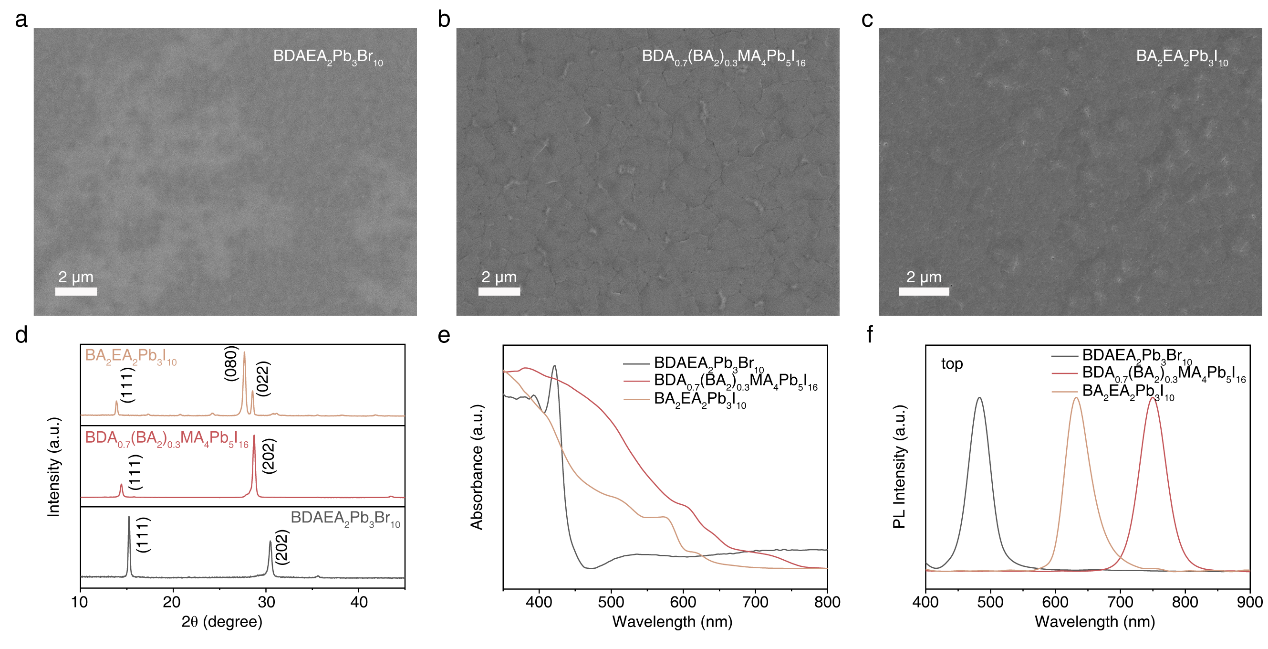


**Figure S39.** Top-view SEM images of the a) BDAEA_2_Pb_3_Br_10_, b) BDA_0.7_(BA_2_)_0.3_MA_4_Pb_5_I_16_, and c) BA_2_EA_2_Pb_3_I_10_ films. d) XRD, e) Abs, and f) PL spectra excited from top side of three quasi-2D MHP films.


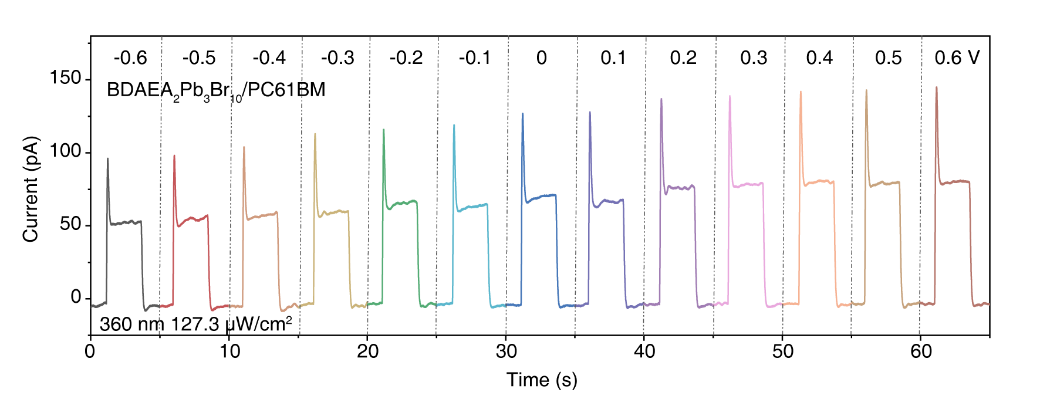


**Figure S40.** The zero bias photoresponses of the BDA_0.7_(BA_2_)_0.3_MA_4_Pb_5_I_16_ film-based PD towards 360 nm laser after been applying with different ferroelectric polarization voltages.


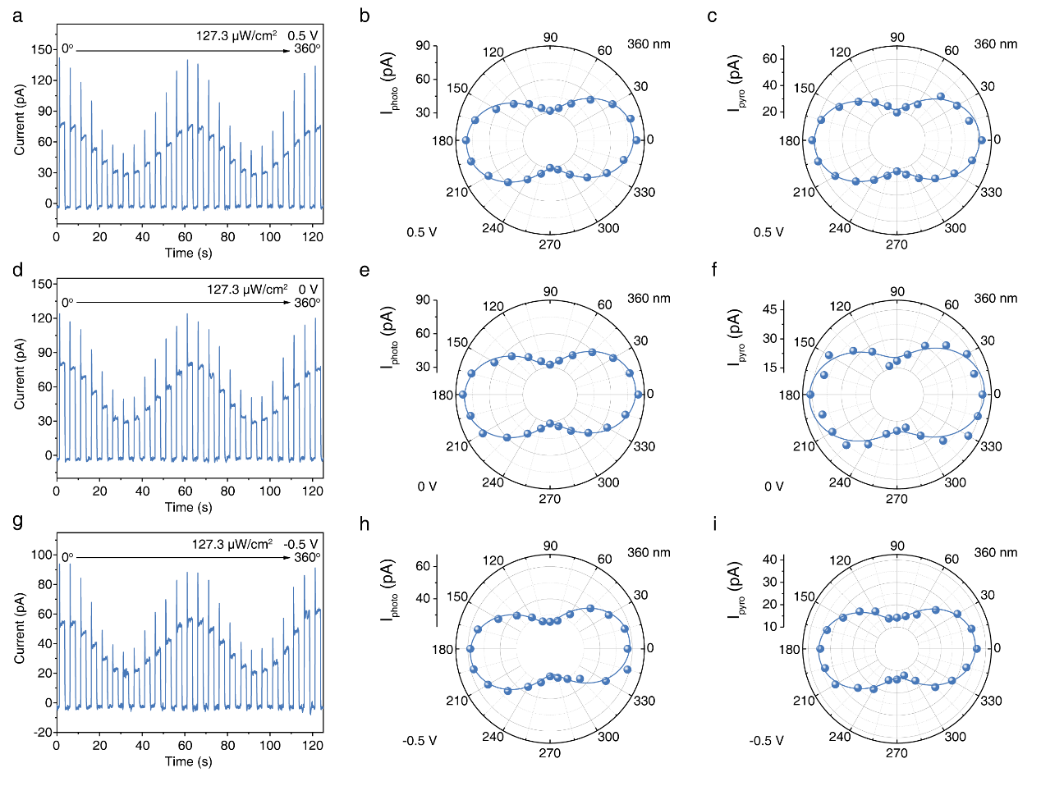


**Figure S41.** Photoresponses of the BDAEA_2_Pb_3_Br_10_ film-based PDs under 360 nm laser with diﬀerent polarization angles at zero bias voltage after been applying with a) 0.5 V, d) 0 V, and g) -0.5 V ferroelectric polarization voltage. Polar diagrams of the polarization-sensitive I_photo_ and I_pyro_ at zero bias for b-c) 0.5 V, e-f) 0 V, and h-i) -0.5 V ferroelectric polarization voltage conditions.


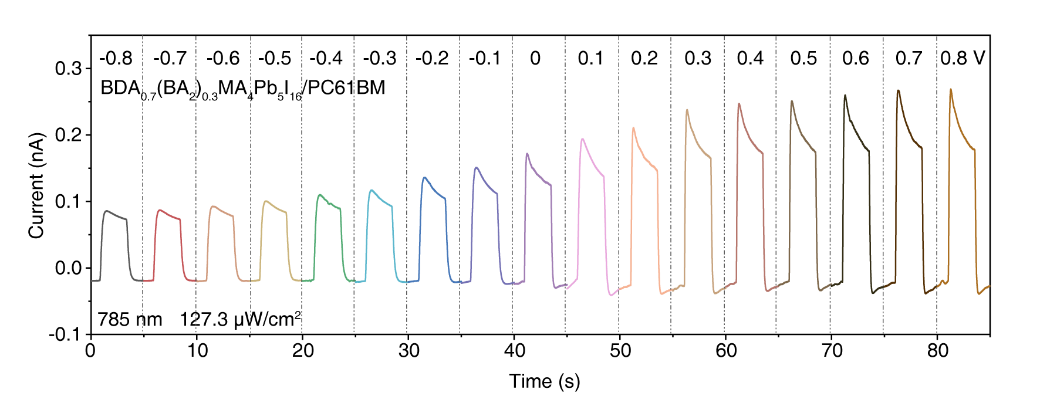


**Figure S42.** The zero bias photoresponses of the BDA_0.7_(BA_2_)_0.3_MA_4_Pb_5_I_16_ film-based PD towards 785 nm laser after been applying with different ferroelectric polarization voltages.


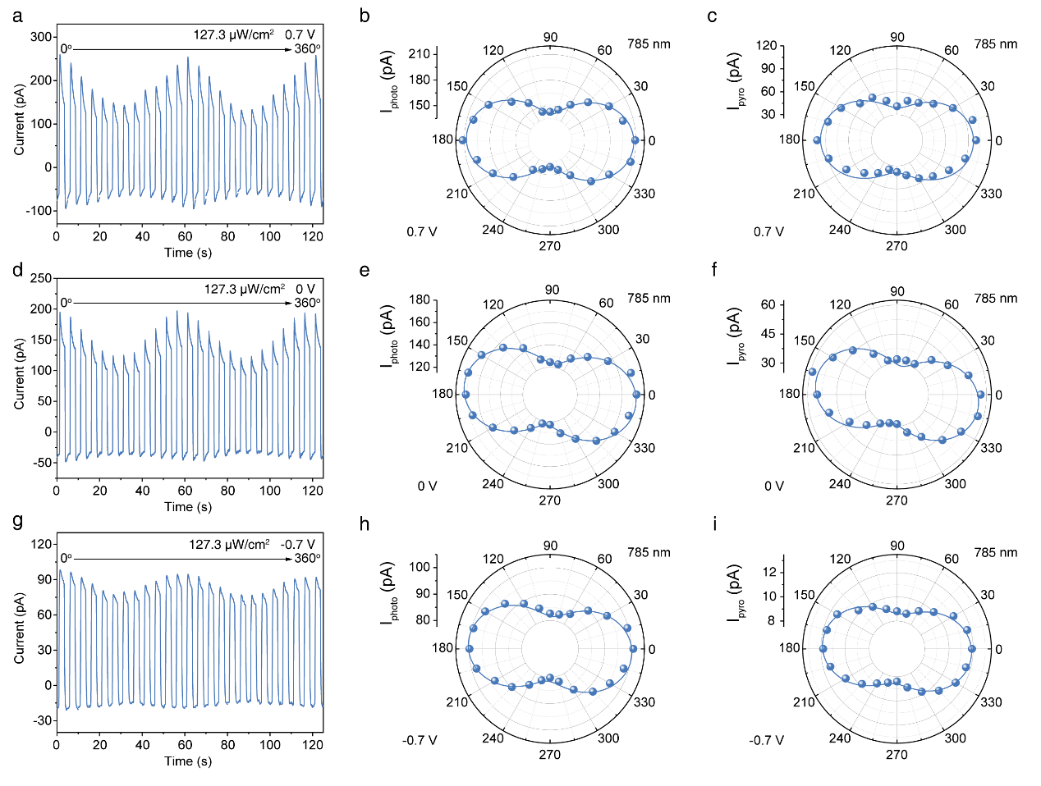


**Figure S43.** Photoresponses of the BDA_0.7_(BA_2_)_0.3_MA_4_Pb_5_I_16_ film-based PDs under 785 nm laser with diﬀerent polarization angles at zero bias voltage after been applying with a) 0.7 V, d) 0 V, and g) -0.7 V ferroelectric polarization voltage. Polar diagrams of the polarization-sensitive I_photo_ and I_pyro_ at zero bias for b-c) 0.7 V, e-f) 0 V, and h-i) -0.7 V ferroelectric polarization voltage conditions.


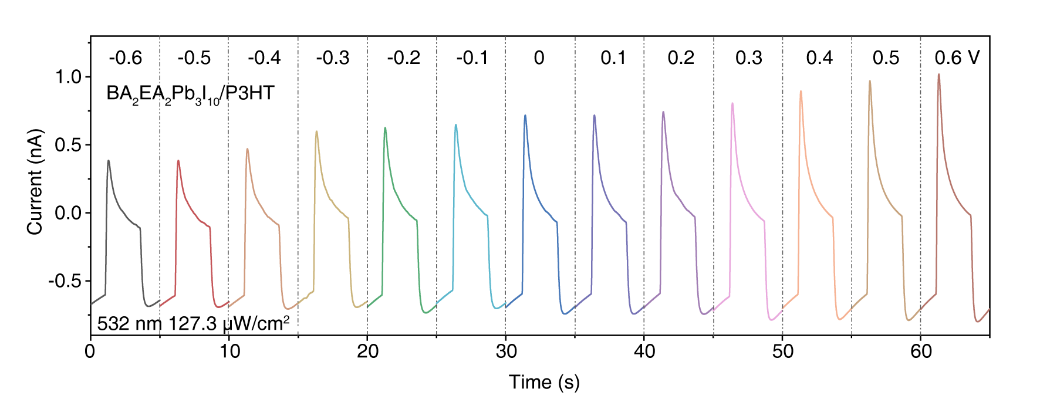


**Figure S44.** The zero bias photoresponses of the BA_2_EA_2_Pb_3_I_10_ film-based PD towards 532 nm laser after been applying with different ferroelectric polarization voltages.


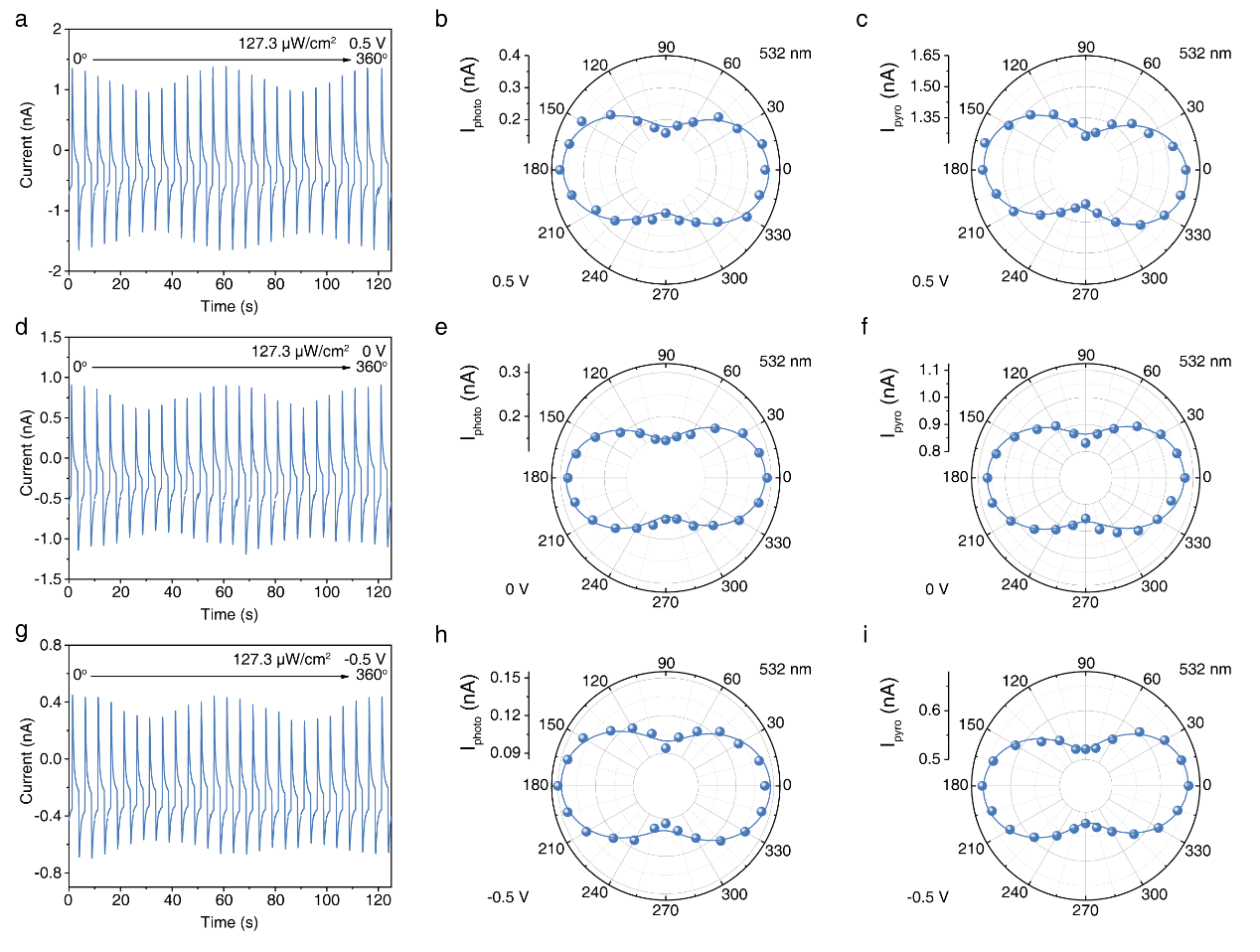


**Figure S45.** Photoresponses of the BA_2_EA_2_Pb_3_I_10_ film-based PDs under 532 nm laser with diﬀerent polarization angles at zero bias voltage after been applying with a) 0.5 V, d) 0 V, and g) -0.5 V ferroelectric polarization voltage. Polar diagrams of the polarization-sensitive I_photo_ and I_pyro_ at zero bias for b-c) 0.5 V, e-f) 0 V, and h-i) -0.5 V ferroelectric polarization voltage conditions.


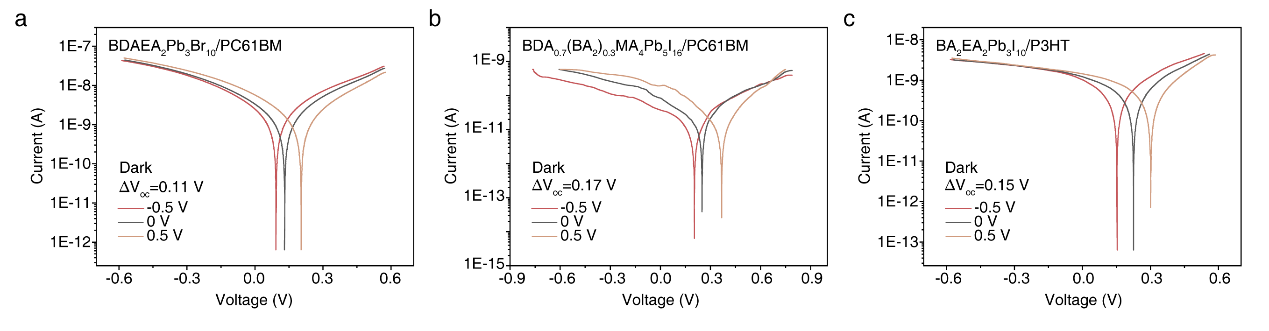


**Figure S46.** Dark *I-V* curves of a) BDAEA_2_Pb_3_Br_10_, b) BDA_0.7_(BA_2_)_0.3_MA_4_Pb_5_I_16_, and c) BA_2_EA_2_Pb_3_I_10_ films heterojunction-based polarized PDs at three different ferroelectric polarization voltages.

**
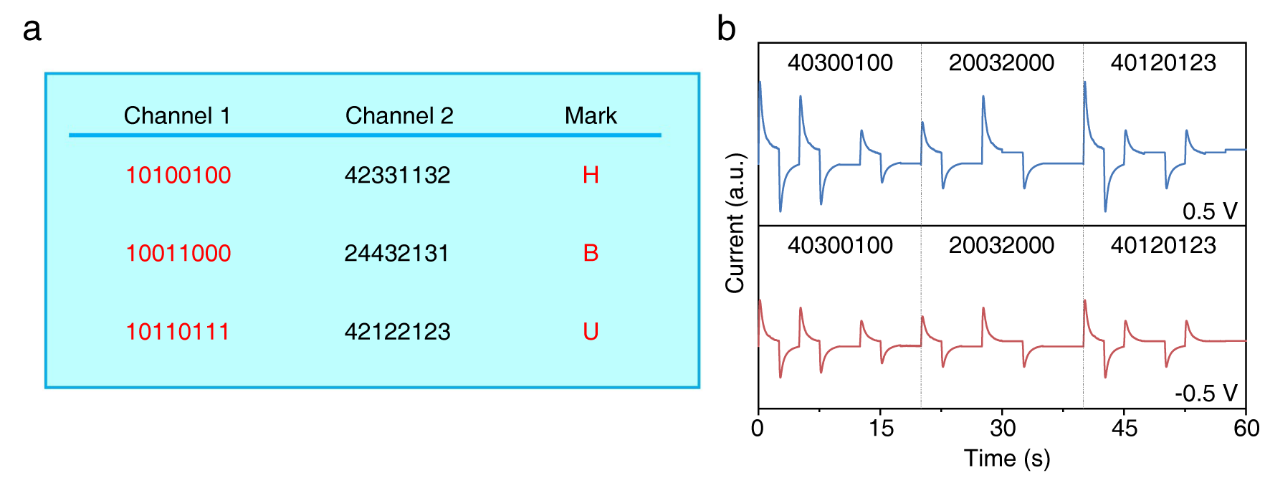
**

**Figure S47.** a) Diagram of the optical signal (HBU, 10100100101100110110111) and polarization signal from the transmitter (Define 0^o^ as 4, 30^o^ as 3, 60^o^ as 2, and 90^o^ as 1). b) When the polarization voltage is 0.5 V (as shown in the figure above) and -0.5 V (as shown in the figure below), the encrypted signal photocurrent is output.

**
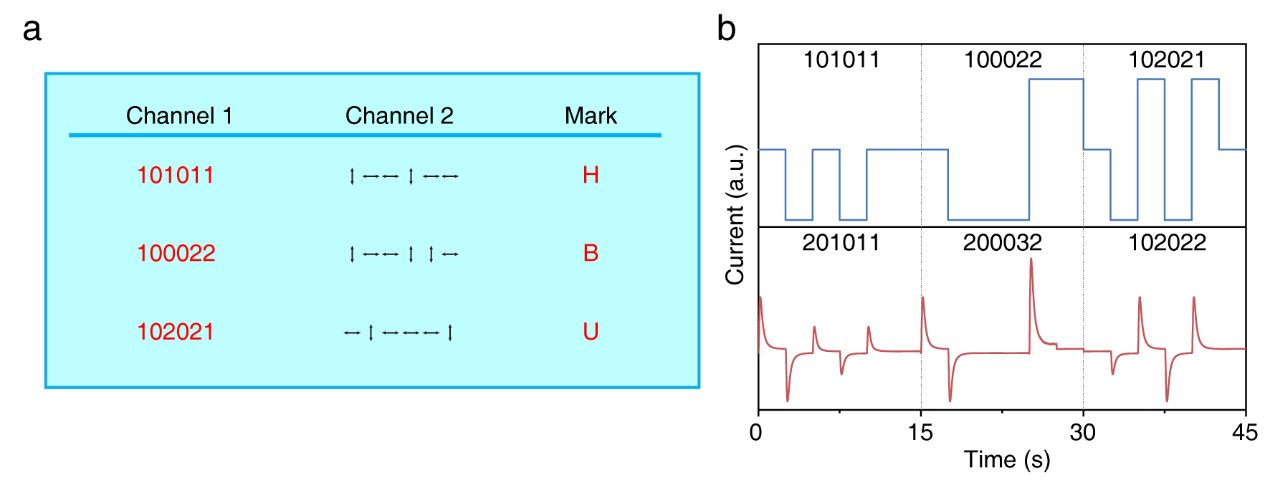
**

**Figure S48.** a) Diagram of the optical signal (HBU, 101011100022102021) and polarization signal from the transmitter, with horizontal arrows for 90° polarized light and vertical arrows for 0° polarized light. b) When only channel 1 is working, the corresponding photocurrent of the encrypted command (as shown in the figure above), and when both channels are running simultaneously, the output encrypted signal photocurrent (as shown in the figure below).


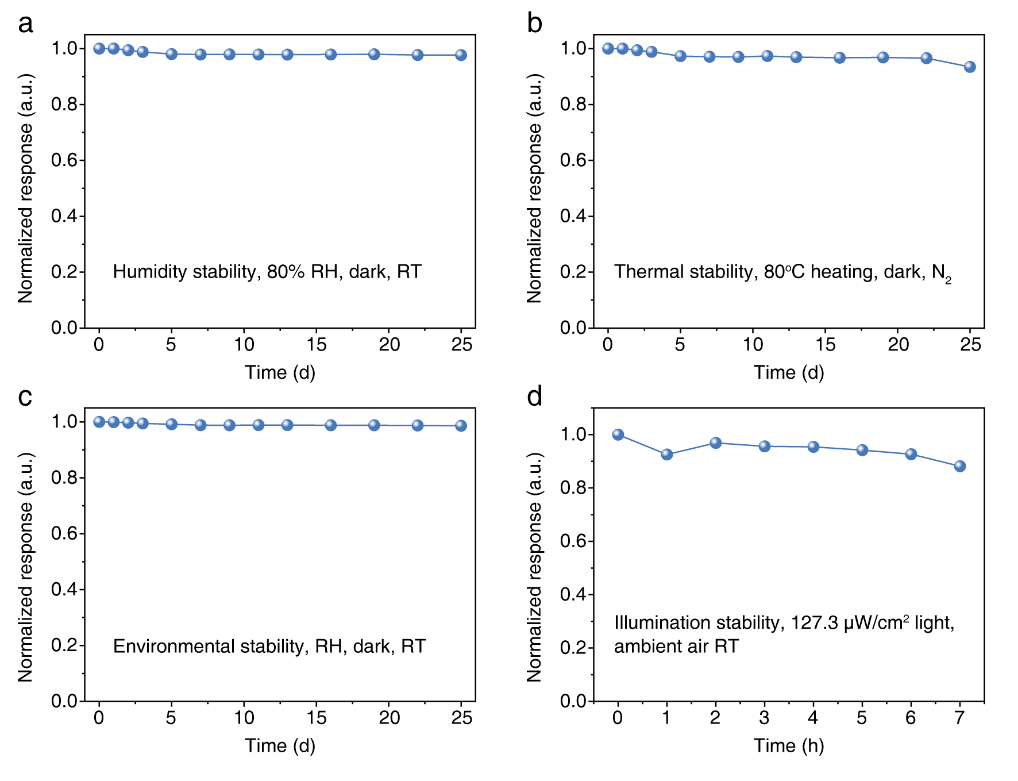


**Figure S49.** a) Humidity stability of 5% Ce^3+^ doped PD in the 80 ± 5% humidity at RT. b) Thermal stability of 5% Ce^3+^ doped PD under continuous heating at 80^o^C in a N_2_-filled glovebox. c) Environmental stability of 5% Ce^3+^ doped PD at room temperature and air humidity. d) Illumination stability of 5% Ce^3+^ doped PD under continuous 320 nm laser illumination (127.3 μW cm^-2^) in 30 ± 5% humidity at RT.


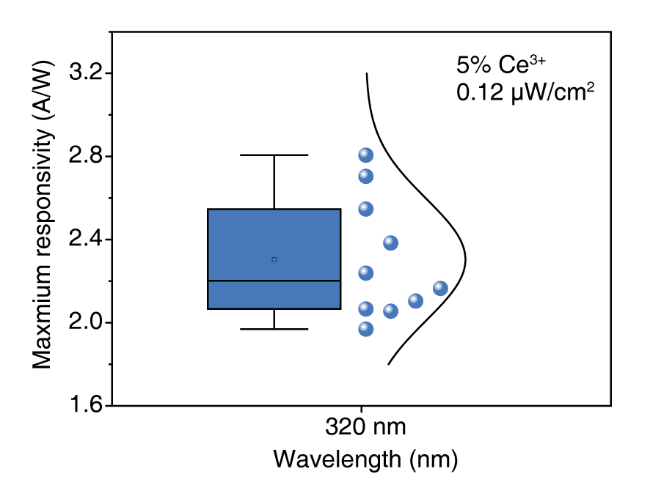


**Figure S50.** The performance statistics of 5% Ce^3+^ PDs under 320 nm, 0.12 μW cm^-2^ laser.
